# Steady state analysis of influx and transbilayer distribution of ergosterol in the yeast plasma membrane

**DOI:** 10.1186/s12976-019-0108-2

**Published:** 2019-08-15

**Authors:** Daniel Wüstner

**Affiliations:** Department of Biochemistry and Molecular Biology, VILLUM Center for Bioanalytical Sciences, University of Southern Denmark, Campusvej 55, DK-5230 Odense M, Denmark

**Keywords:** Sterol, Cholesterol, Ergosterol, Flip-flop, Plasma membrane, Flux, Non-equilibrium, Steady state, Esterification, Endoplasmic reticulum

## Abstract

**Background:**

The transbilayer sterol distribution between both plasma membrane (PM) leaflets has long been debated. Recent studies in mammalian cells and in yeast show that the majority of sterol resides in the inner PM leaflet. Since sterol flip-flop in model membranes is rapid and energy-independent, a mechanistic understanding for net enrichment of sterol in one leaflet is lacking. Import of ergosterol in yeast can take place via the ABC transporters Aus1/Pdr11 under anaerobic growth conditions, eventually followed by rapid non-vesicular sterol transport to the endoplasmic reticulum (ER). Little is known about how these transport steps are dynamically coordinated.

**Methods:**

Here, a kinetic steady state model is presented which considers sterol import via Aus1/Pdr11, sterol flip-flop across the PM, bi-molecular complex formation and intracellular sterol release followed by eventual transport to and esterification of sterol in the ER. The steady state flux is calculated, and a thermodynamic analysis of feasibility is presented.

**Results:**

It is shown that the steady state sterol flux across the PM can be entirely controlled by irreversible sterol import via Aus1/Pdr11. The transbilayer sterol flux at steady state is a non-linear function of the chemical potential difference of sterol between both leaflets. Non-vesicular release of sterol on the cytoplasmic side of the PM lowers the attainable sterol enrichment in the inner leaflet. Including complex formation of sterol with phospholipids or proteins can explain several puzzling experimental observations; 1) rapid sterol flip-flop across the PM despite net sterol enrichment in one leaflet, 2) a pronounced steady state sterol gradient between PM and ER despite fast non-vesicular sterol exchange between both compartments and 3) a non-linear dependence of ER sterol on ergosterol abundance in the PM.

**Conclusions:**

A steady state model is presented that can account for the observed sterol asymmetry in the yeast PM, the strong sterol gradient between PM and ER and threshold-like expansion of ER sterol for increasing sterol influx into the PM. The model also provides new insight into selective uptake of cholesterol and its homeostasis in mammalian cells, and it provides testable predictions for future experiments.

## Background

**Fig. 1:**
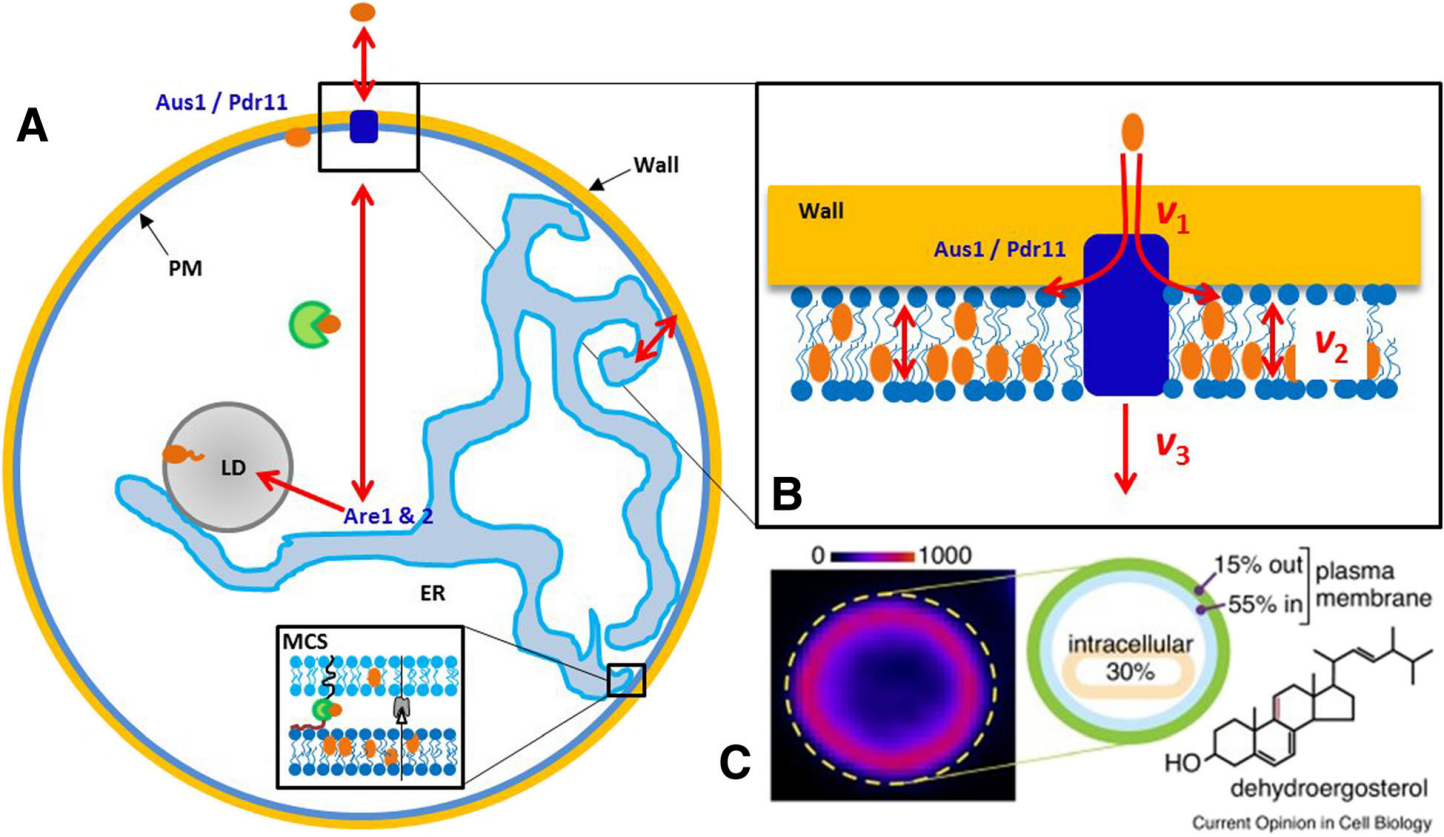
Sterol import into yeast under anaerobic growth conditions. The two ABC transporters, Aus1 and Pdr11 import sterol (brown ellipses) into the plasma membrane (PM; **a**). Once in the PM, sterol can reach the endoplasmic reticulum (ER) via non-vesicular transport (red arrows) bound to sterol transfer proteins through the cytosol or at membrane contact sites (MCS). In the ER, sterol can be esterified by Are1/2 and stored in lipid droplets (LD). **b** Transport steps considered in the model in Eq. 1; sterol import by Aus1/Pdr11 with rate *v*_1_, sterol flip-flop with rate *v*_2_ and non-vesicular sterol transport with rate *v*_3_. **c** sterol in the PM visualized using dehydroergosterol (DHE) showing that most sterol resides in the inner compared to the outer PM leaflet

Cells require sterols for growth and acquire these molecules from the extracellular medium or by de novo synthesis. Sterol transport in the bloodstream and uptake by cells is achieved with the participation of low density lipoprotein (LDL) receptors. While much information has been gathered about these processes, transport of sterols within cells is poorly understood [1–3]. Mammalian cells internalize cholesterol either by receptor mediated endocytosis of LDL or by selective sterol uptake processes. Selective sterol influx into the plasma membrane (PM) from circulating high density lipoprotein (HDL) is mediated by scavenger receptor BI (SR-BI) in the liver and gonads. Sterol import into the yeast PM takes place under anaerobic conditions and is mediated by ATP-dependent transport via the ABC transporters Aus1 and Pdr11 and potentially other transporters. In both, mammalian and yeast cells, intracellular sterol transport includes a non-vesicular pathway. In non-vesicular transport, sterol molecules are extracted from the cytoplasmic face of a ‘donor’ membrane by a carrier protein and off-loaded at the ‘acceptor’ compartment [4, 5]. Since sterols are very hydrophobic molecules, the thermodynamic sterol partition into the aqueous cytoplasm will be very low (on the order of 1–5∙10^6^ in favor of the membrane) [6]. Due to its hydrophobicity and membrane condensing capacity, the activation energy for cholesterol to leave a lipid bilayer, *E*_A_, is very high (*E*_A_ ≈ 75 kJ/mol, that is 30fold *k*_b_∙T) [6, 7]. Release rate constants have been directly measured from liposomes for dehydroergosterol (DHE), a fluorescent sterol differing from the natural yeast sterol ergosterol only by having one additional double bond [6]. The values were in the range of *k* ≈ 1∙10^− 3^ s^− 1^ for POPC (t_1/2_ = 11.6 min) over 6.4∙10^− 4^ s^− 1^ (t_1/2_ = 18.1 min) for a 1:1 mixture of POPC-cholesterol to 5∙10^− 5^ s^− 1^ (t_1/2_ = 3.85 h) for liposomes made of sphingomyelin-cholesterol, respectively [6]. Thus, sterol desorption depends on the bilayer lipid composition and is rate-limiting for overall passive sterol exchange between membranes [5, 8]. In contrast, transport of ergosterol between PM and ER in intact living yeast cells takes place with a half time of t_1/2_ = 4–10 min [9]. Thus, cells must have efficient mechanisms to speed up sterol exit from a bilayer for efficient non-vesicular sterol transport between organelle membranes. One possibility to guarantee intracellular non-vesicular sterol transport despite the low water solubility of sterols is the expression of sterol transport proteins (STPs) which lower *E*_A_ for sterol release from a membrane [5, 8]. Such STPs can be soluble cytoplasmic proteins which lower the free energy cost of sterol solvation by accommodating the released sterol in a hydrophobic binding pocket. They can also be membrane proteins, containing two structural motifs, which anchor them simultaneously in the PM and in the ER, thereby creating close membrane contact sites (MCS) that may enhance the efficiency of STPs by confining them to a narrow space (Fig. 1) [10, 11]. The yeast *S. cerevisiae* contains ergosterol as main sterol, and many homologs to mammalian STPs have been discovered in this well-established model organism [12]. Recently discovered STPs include the oxysterol binding protein (OSBP) family, StART-like StARD4, Aster and GRAM proteins in mammalian cells as well as the related OSBP homologs (Osh) proteins and Lam proteins in yeast. These proteins have been implicated in non-vesicular sterol transport to the endoplasmic reticulum (ER), endosomes and to lipid droplets (LDs). Thus, allocation of sterol to the inner PM leaflet after selective uptake is necessary for its subsequent non-vesicular transport to various intracellular sites.

Rapid non-vesicular sterol transport between membranes raises the question, how cells can maintain strong sterol gradients between organelles at steady state [8, 10, 13]. It must mean that certain mechanisms ensure equal chemical potential of cholesterol in different cellular membranes despite varying concentrations. This, in turn, invokes membrane dependent activity coefficients due to specific interactions of sterols with other membrane components [8, 10, 13, 14]. Several observations made on regulation of cholesterol uptake and metabolism in mammalian cells can be rationalized with a model assuming two pool of cholesterol in the PM; active cholesterol is able to move freely between membranes by non-vesicular transport, while a second cholesterol pool forms stoichiometric complexes with other membrane components, such as phospho- and sphingolipids bearing saturated acyl chains [14–16]. In this model, cholesterol abundance in the PM sets a threshold for a sudden increase of active cholesterol once the capacity of the PM to form stoichiometric complexes is exceeded. Above this threshold, rapid flux of active cholesterol from the PM to the ER takes place within 10–15 min until cholesterol’s chemical potential in both membranes is equal again [10]. The sudden expansion of the sterol pool in the ER triggers feedback responses like activation of cholesterol esterification by acyl-Coenzyme A acyl transferase (ACAT) and shutdown of cholesterol synthesis via inhibition of the SCAP/INSIG/SREBP transcription factor complex [17–19]. This model has been also invoked to explain the strong ergosterol gradient between PM and ER in yeast despite continuous rapid non-vesicular sterol equilibration taking place in ca. 10 min [9, 20, 21]. It is not known how eventual sterol complex formation is kinetically regulated during sterol uptake and flip-flop in the PM, and no attempt has been made, so far, to incorporate sterol complex formation in a steady state model of cellular sterol transport.

Sterol transport in yeast can be conveniently studied using live-cell imaging of the fluorescent DHE (Fig. 1c). DHE is a natural yeast sterol with comparable biophysical properties as ergosterol and cholesterol in model and cell membranes [22, 23]. DHE is like ergosterol taken up by yeast under anaerobic growth conditions, and its import into the yeast PM depends strictly on Aus1/Pdr11 [21, 24, 25]. In cells, lacking functional Aus1/Pdr11, DHE is not inserted into the PM but gets stuck in the cell wall from where it can be extracted using organic solvents [24]. Thus, these ABC transporters are necessary for insertion of sterol from the cell wall into the PM (see Fig. 1a, b). After shifting to aerobic conditions, DHE is internalized and partly esterified by sterol acyltransferases Are1 and 2 and deposited in lipid droplets (LD; Fig. 1a), exactly as ergosterol in wild-type cells [26, 27]. This can be quantified in kinetic imaging experiments relative to known organelle markers [27, 28]. Transport-coupled esterification of DHE is monitored in parallel [29, 30]. Recently, we developed an assay for measuring the sterol transbilayer distribution in the PM of living yeast cells [31]. We used sterol-auxotroph yeast cells lacking Hem1 (Δ*hem1* cells), which makes them unable to use oxygen in ergosterol synthesis such that they can use DHE as only sterol source for growth and survival [27, 31]. In Δ*hem1* cells, ergosterol synthesis is eliminated and most DHE stays in the PM (Fig. 1c and [4, 31]). This allows for quantification of the transbilayer distribution of DHE in the PM using side-specific quenchers, which showed that up to 80% of DHE in the PM resides in its inner leaflet [31]. This asymmetric sterol distribution did not depend on metabolic energy, subcortical actin or a PM proton gradient. Sterol asymmetry across the PM required long-chain sphingolipids in the PM and was strongly reduced in cells lacking Drs2, a P-type ATPase transporting phosphatidylserine (PS) to the cytoplasmic leaflet of the PM and Golgi apparatus [31]. Since DHE and other sterols rapidly traverse model lipid membranes [32, 33], it is not clear how such a strong transbilayer sterol gradient can be established and maintained in the PM of living cells. Similarly, exactly how cellular sterol import, sterol distribution between the two PM leaflets and non-vesicular intracellular sterol transport is coordinated, is not known.

Here, we have developed a mathematical steady state model for sterol import, sterol flip-flop across the yeast PM and cytoplasmic non-vesicular sterol transport. We derive analytical expressions, investigate their implications and suggest how they can be used for making concrete predictions in future experiments.

This paper is organized as follows; first, we introduce an irreversible model of sterol import with one sterol pool in each leaflet. We analyze the thermodynamics of the steady state flux for this model afterwards. Second, we extend this model by assuming reversible sterol import. Subsequently, a 2-pool model is introduced which accounts for sterol complex formation in the PM. This model is first discussed in a linearized version to develop key principles including sterol transport between PM and ER as well as sterol esterification in the ER. Next, this model is extended to account for biomolecular stoichiometric complex formation of sterol in the PM and ER, and the implications of this extension on sterol transport are discussed. Finally, results and predictions of our model are summarized and their implications for future experimental studies are discussed. Although developed for ergosterol transport in yeast, the model bears important implications for trafficking of cholesterol in mammalian cells. Accordingly, we refer also to cholesterol homeostasis in mammalian, wherever appropriate.

## Methods

All calculations were carried out manually or with help of the Mathematica software (Wolfram Research Oxfordshire, UK). Analytical solutions were used for simulations using SigmaPlot (Systat Inc., Erkrath, Germany), which was also used for generating figures.

## Results

### Model of sterol transport in yeast with one sterol pool in each PM leaflet

#### Irreversible sterol import via Aus1/Pdr11 – steady state and control analysis

As a starting point for our discussion, we consider a simple model of sterol import in Δ*hem1* cells due to active transport by Aus1/Pdr11 with rate *v*_1_, sterol flip-flop with rate *v*_2_ and sterol release from the inner PM leaflet to the ER with rate *v*_3_, where sterol is esterified (see Fig. 1b). Yeast cells are surrounded by a cell wall containing highly hydrated carbohydrates across which sterols cannot diffuse passively. Instead, sterol import requires the ABC transporters Aus1/Pdr11, which mediate transport across the cell wall and likely direct sterol insertion into the PM outer leaflet [25, 4]. We assume that any sterol released from the PM is transported to the ER, where it is esterified by Are1 and Are 2 and stored as esters in LDs (Fig. 1a). This esterification process replenished by non-vesicular sterol release removes sterols from the PM and acts as permanent sink in our model. However, in this first modeling step, only the steady state flux across the PM is considered, while sterol arrival in the ER and sterol esterification are not explicitly taken into account, yet. These processes only enter the model at this stage via their effect on removing sterol released on the inner PM leaflet into the cytosol. This sterol removal is described by rate *v*_3_. Previous modeling work has shown that non-vesicular sterol transport by STPs is likely not limited by intracellular diffusion of sterol-protein complexes but rather by sterol pick up from the bilayer, at least in a physiologically relevant concentration range in small cells, like yeast [5]. Thus, concentration gradients of sterol-STP complexes in the cytoplasm can be ignored allowing for a simple ansatz using ordinary differential equations (ODEs) in our model. We have also not explicitly accounted for any volume effects to avoid extensive need for parametrization of our model. Accordingly, substance amounts and concentrations are often used synonymously. We have focused on the key features of coupled sterol import, transbilayer migration and cytosolic sterol release, allowing for a simple steady state description of the transport process. Being aware of possible non-ideal mixing of membrane sterols with phospholipids giving rise to membrane dependent activity coefficient of sterols, we nevertheless start out with a simple description using ideal solution theory. This is to obtain first insight into the underlying kinetic principles and steady state properties. Non-ideal behavior via sterol-phospholipid complexes is considered subsequently by extending the simpler kinetic model. Finally, we employ first-order kinetics by assuming that cytoplasmic STPs are not consumed in the sterol transfer reaction (i.e., k_3_ = k_3_*∙[STP], where the concentration of STPs, [STP] = constant). The extracellular sterol concentration, i.e. sterol in the medium, *S*_0_, is kept constant (clamped). This is realized in experiments by providing an excess supply of a tracer sterol (e.g. DHE) in Tween and oleic acid for 24-48 h, during which a steady state of sterol flux across the PM is established [31]. Alternatively, low amounts of sterol could be steadily supplied in a microfluidic device, thereby ensuring constant DHE levels in the medium. This model corresponds to the following kinetic scheme:

1 $$ {S}_0\overset{v_1}{\to }{S}_1\overset{v_2}{\leftrightarrow }{S}_2\overset{v_3}{\to } $$

The corresponding differential equations for sterol in the outer PM leaflet (*S*_1_) and the inner PM leaflet (*S*_2_) read with rates *v*_1_ = *k*_1_ ⋅ *S*_0_, *v*_2_ = *k*_2_ ⋅ *S*_1_ − *k*_−2_ ⋅ *S*_2_ and *v*_3_ = *k*_3_ ⋅ *S*_2_, respectively:

2a, b $$ {\displaystyle \begin{array}{l}\frac{dS_1}{dt}={v}_1-{v}_2={k}_1\cdot {S}_0-{k}_2\cdot {S}_1+{k}_{-2}\cdot {S}_2\\ {}\frac{dS_2}{dt}={v}_2-{v}_3={k}_2\cdot {S}_1-\left({k}_{-2}+{k}_3\right)\cdot {S}_2\end{array}} $$

The system (or Jacobian) matrix for the equations in (2a, b) contains the rate constants and has determinant |*A*| = *k*_2_ ⋅ *k*_3_ > 0, indicating that we have a non-trivial steady state. As it is a linear system with negative real eigenvalues of *A*, this steady state is a stable fix point. The steady state amount of sterol in the outer and inner leaflet of the PM can be determined from Eq. 2a, b using Cramer’s rule to:

3a $$ \overline{S_1}=\frac{\left|{A}_1\right|}{\left|A\right|}=\frac{k_1\cdot {S}_0\cdot \left({k}_{-2}+{k}_3\right)}{k_2\cdot {k}_3}=\frac{v_1\cdot \left({k}_{-2}+{k}_3\right)}{k_2\cdot {k}_3} $$

3b $$ \overline{S_2}=\frac{\left|{A}_2\right|}{\left|A\right|}=\frac{k_1\cdot {k}_2\cdot {S}_0}{k_2\cdot {k}_3}=\frac{k_1\cdot {S}_0}{k_3}=\frac{v_1}{k_3} $$

Thus, the sterol amount in the inner leaflet at steady state is completely independent of the flip-flop rates, but solely determined by sterol insertion into and sterol release from the PM in this model. Using these expressions in (2a, b), we can find the steady state flux as:

4a, b $$ {\displaystyle \begin{array}{l}\overline{v_2}={k}_2\cdot \overline{S_1}-{k}_{-2}\cdot \overline{S_2}={v}_1\\ {}\overline{v_3}={k}_3\cdot \overline{S_2}={v}_1\end{array}} $$

Thus, the steady state flux through the PM is entirely determined by the influx via the ABC transporters Aus1/Prd11 in this model. In other words, the details of the sterol flip-flop mechanism do not impact the steady state flux of sterol from the medium into the cell. They do affect the kinetics of sterol import, though.

Based on these results, lets define the influx more properly. A reasonable assumption is that of an irreversible Michaelis-Menten-type kinetics, similar to glucose import by the GLUT transport system [34]:

5 $$ {S}_0+E\overset{m_{1,-1}}{\leftrightarrow }{ES}_0\overset{m_2}{\to }{S}_1+E $$

Here, *E* is the amount of Aus1/Pdr11, acting as an enzyme for sterol import; *m*_1_ and *m*_− 1_ are the association and dissociation rate constants for sterol in the medium (*S*_0_) and Aus1/Pdr11 at the outer side of the cell, respectively. The catalyzed insertion of sterol into the outer leaflet is modeled with rate constant *m*_2_. This leads with the classical quasi-steady state assumption for the *ES*_0_ complex to the well-known hyperbolic Michaelis-Menten type law for sterol import rate (*v*_1_^MM^):

6 $$ {v}_1^{MM}=\frac{v_{\mathrm{max}}\cdot {S}_0}{k_M+{S}_0} $$

Here, *v*_max_ is the maximal transport rate of the Aus1/Pdr11 transport system (=*m*_2_·*E*_T_, with E_T_ being the total amount of Aus1/Pdr11 transporters in the PM), and the Michaelis-Menten constant is ${k}_M=\frac{m_{-1}+{m}_2}{m_1}$. With that, we can express the steady state flux into the cell as function of the kinetic parameters of the Aus1/Pdr11 transport system. The hyperbolic kinetics of sterol uptake by Aus1/Pdr11 can be linearized for two extreme cases:

##### Low-substrate range

In the low-substrate regime, i.e., for low external sterol, Eq. 6 can be linearized according to:

7 $$ {v}_{1, lin}^{MM}={\left.\frac{\partial {v}_1^{MM}}{\partial {S}_0}\right|}_{S_0\to 0}\cdot {S}_0=\frac{v_{\mathrm{max}}}{k_M}\cdot {S}_0={k}_1\cdot {S}_0={v}_1 $$

Thus, for low amounts of sterol in the medium, a linearization of the irreversible import model recovers the rate constant *k*_1_ from the differential equation system in Eq. 2a, b with ${k}_1\cdot {S}_0=\frac{v_{\mathrm{max}}}{k_M}\cdot {S}_0=\frac{m_l\cdot {E}_T}{k_M}\cdot {S}_0$. This situation could apply when using a microfluidic device to ensure a constant but low supply of ergosterol or its fluorescent analogue DHE. One could also use highly fluorescent tagged analogues of cholesterol, like nitrobenzoxadiazole (NBD)-tagged cholesterol, for which a steady supply of trace amounts would be sufficient to achieve sufficient staining of cells [35, 36]. Finally, one could express mutants of Aus1/Pdr11 or use inhibitors, which both impact the binding of sterol substrate to the transporters, thereby increasing their *k*_M_ values and shifting the linear regime of the hyperbolic Michaelis-Menten kinetics to higher substrate values. For this linear substrate-transport relationship, we ask how an infinitesimal change in the enzyme parameters of Aus1/Pdr11 will affect the steady state flux of sterol into the cell. To answer that, we use the fact that at steady state, we have ${v}_1={k}_1\cdot {S}_0={\overline{v}}_2={\overline{v}}_3={\overline{v}}_1=J$ and calculate the flux control coefficients, a measure quantifying the impact of infinitesimal parameter changes on the steady state flux [37]. The only non-trivial flux control coefficient is:

8 $$ {C}_{k_1}^J=\frac{k_1}{J}\cdot \frac{\partial J}{\partial {k}_1}=1 $$

All other flux control coefficients are zero. Thus, the total control about sterol flux into the cell lies in the ATP-driven sterol import process in our model, and a small change in the activity or abundance of Aus1/Pdr11 should directly translate into a proportional change in sterol import flux and sterol abundance in both leaflets. This conclusion, however, is only valid, as long as we consider linear kinetics and infinitesimal changes in the parameter values for the import process.

In a similar manner, we can calculate concentration control coefficients, generally defined as [37]:

9 $$ {C}_k^{S_i}={\left(\frac{v_k}{S_i}\frac{\Delta\;{S}_i}{\Delta\;{v}_k}\right)}_{\Delta\;{v}_k\to 0}=\frac{v_k}{S_i}\frac{\mathit{\partial}\;{S}_i/\mathit{\partial}\;{p}_k}{\mathit{\partial}\;{v}_k/\mathit{\partial}\;{p}_k}=\frac{\mathit{\partial}\;\ln {S}_i}{\mathit{\partial}\;\ln {v}_k} $$

For sterol in the outer leaflet, changing the import rate constant, for example by altering the catalytic activity or abundance of Aus1/Pdr11 or by slightly changing the sterol amount in the medium will give:

10 $$ {C}_J^{{\overline{S}}_1}=\frac{J}{{\overline{S}}_1}\cdot \frac{\partial {\overline{S}}_1}{\partial J}=\frac{k_1\cdot {S}_0\cdot {k}_2\cdot {k}_3}{k_1\cdot {S}_0\cdot \left({k}_{-2}+{k}_3\right)}\frac{\left({k}_{-2}+{k}_3\right)}{k_2\cdot {k}_3}=1 $$

Similarly, one can show that the control over the steady state amount of sterol in the inner leaflet is entirely set by the sterol import process, i.e., one gets ${C}_J^{{\overline{S}}_2}=$ 1. Thus, total steady state amounts of sterol in the outer and inner leaflet, respectively, follow changes in the sterol import step in a concerted manner.

##### High-substrate range

In the high-substrate range (i.e., for *S*_0_ → ∞), Eq. 7 gives *v*_1_^MM^ = *v*_max_, i.e., sterol excess in the medium would make that the Aus1/Pdr11 transport system is always saturated and works under its maximal capacity. In that situation, sterol import becomes independent of the actual sterol concentration in the medium. Recently, we carried out ergosterol uptake experiments using its close analog DHE in *hem1*Δ cells [31]. Experiments on uptake of the ergosterol analogue DHE had been conducted in log-phase growing yeast with OD600 around 0.75 corresponding to 1.16∙10^7^ cells/ml. To these cells 20 μg/ml DHE in Tween had been added, which equals a concentration of 50.68 μM [31]. Thus, translated into our model, we have *S*_0_ = 50.68 μM. After 24-48 h culture, all ergosterol in the cells had been replaced by DHE [31]. A yeast cell has about 1∙10^8^ ergosterol molecules [20], thus, there are 1.16∙10^15^ DHE molecules/ml incorporated into the cells in the 1-ml suspension, corresponding to 1.926 μM. Accordingly, DHE in the medium is in large (>25fold) excess of DHE in the cells, which corresponds to situation b), in which *v*_1_ = *v*_max_. Thus, uptake becomes independent of DHE abundance in the medium (0. order kinetics), and the steady state flux is entirely set by the maximal capacity of the sterol import system Aus1/Pdr11. This corresponds to a constant (clamped) concentration *S*_0_. Since *v*_max_ = *m*_2_·*E*_T_, the expression level of Aus1/Pdr11 transporters in the PM, E_T_, translates in this case directly into the magnitude of sterol flux across the yeast PM at steady state. There are about 10.5∙10^3^ Aus1 transporters per yeast cell (http://yeastgenome.org/), which corresponds to 12.18∙10^13^ transporters per l, equal to 2.02∙10^− 10^ mol/l. Thus, one can estimate *E*_T_ = 0.2 nmol/l for the DHE uptake experiments carried out previously [4, 31]. Furthermore, the catalytic efficiency of purified and reconstituted Aus1/Pdr11 has been estimated to hydrolysis of 10 ATP molecules per protein per second, thus 0.167 ATP’s per sec [38, 39]. Assuming that the catalytic efficiency is the same in intact cells and that there is a 1:1 stoichiometric coupling between sterol transport and ATP-hydrolysis by these ABC transporters, one gets *m*_2_ = 0.167 transported sterol molecules per sec. Thus, we arrive at *v*_max_ = 0.033 nmol/(l∙s) as the maximal possible capacity of the sterol influx system in our yeast cell culture. Is this a reasonable number? As a test, we ask how long it would take to reach a steady state DHE labeling of 1.926 μM (see above) with this flux magnitude, which gives 1926 nmol/l: 0.033 nmol/(l∙s) =58363,6 s ≈ 16 h. This value is comparable to the 24-h sterol labeling procedure, which we typically used. Thus, we conclude that these numbers are very reasonable.

#### Intracellular sterol release affects the transbilayer sterol distribution in the PM

Exit of sterol from the cytoplasmic leaflet is supposed to be an important control point in setting the sterol content of the ER and thereby regulating overall sterol homeostasis in both, yeast and mammalian cells [13, 14]. Release of cholesterol from the PM of mammalian cells is supposed to be rather slow compared to transbilayer sterol flip flop under normal growth conditions resulting for example in bi-phasic transport to the endocytic recycling compartment in fibroblasts [40]. Acute cholesterol loading, intercalation of membrane active molecules or depletion of sphingolipids can result in a non-linear increase of cholesterol release from the PM and thereby acute expansion of the cholesterol pool in the ER and other organelles [41–44]. In yeast, exit of the fluorescent ergosterol analogue DHE from the PM is low under anaerobic growth conditions but can be enhanced several fold after switching to aerobic growth, likely because newly made ergosterol can replace some DHE in the PM [20, 27, 31]. Here, more DHE is made available for pick up by cytosolic STPs, thereby increasing the rate of non-vesicular transport of DHE from the PM to the ER. In the ER, the sterol get esterified and stored in lipid droplets, which was found to require metabolic energy and activity of the yeast ACAT homologue, Are2 [27]. Thus, in both yeast and mammalian cells, sterol exit from the PM seems to depend on active sterol esterification. In our model of Eq. 1, sterol exit from the PM and its esterification in the ER is summarized by rate v_3_ = k_3_∙S_2_, and a relevant question is, how this rate affects the steady state ratio of sterol in the PM. This reads:

11 $$ \overline{S_2}/\overline{S_1}=\frac{v_1}{k_3}\cdot \frac{k_2\cdot {k}_3}{v_1\cdot \left({k}_{-2}+{k}_3\right)}=\frac{k_2}{k_{-2}+{k}_3}=\frac{q_2}{1+{k}_3/{k}_{-2}}=Q $$

**Fig. 2:**
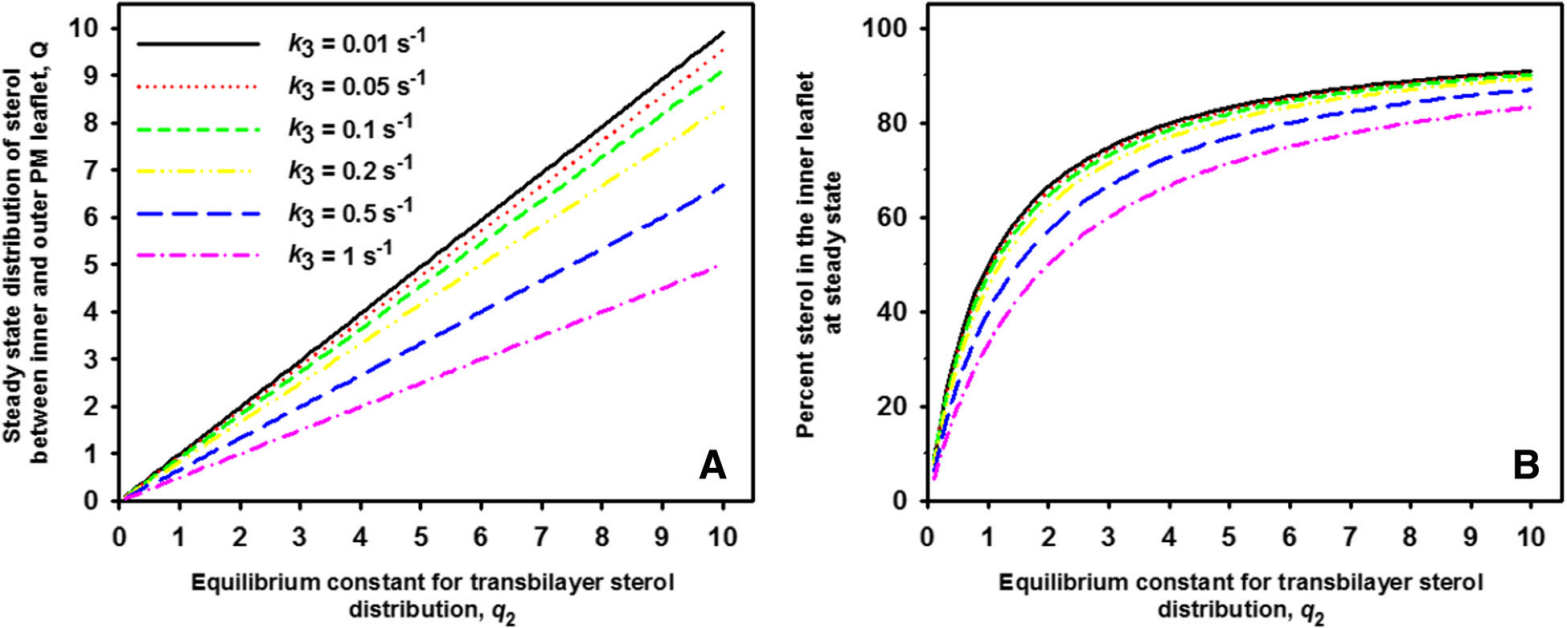
Increasing intracellular sterol release rates decrease the attainable sterol asymmetry in the PM. Steady state transbilayer sterol distribution (**a** ratio Q, Eq. 11) and percent sterol in the inner leaflet (**b**) as function of the passive sterol distribution between both PM leaflets (*q*_2_) for the indicated rate constants of intracellular sterol release (*k*_3_) for the irreversible sterol import model. A ratio Q = 1 corresponds to a symmetric sterol distribution between both leaflets, while Q > 1; Q < 1 means sterol enrichment in the inner and outer leaflet, respectively. Other parameters were *v*_1_ = 1 mol/s and *k*_− 2_ = 1 s^− 1^, respectively

**Fig. 3:**
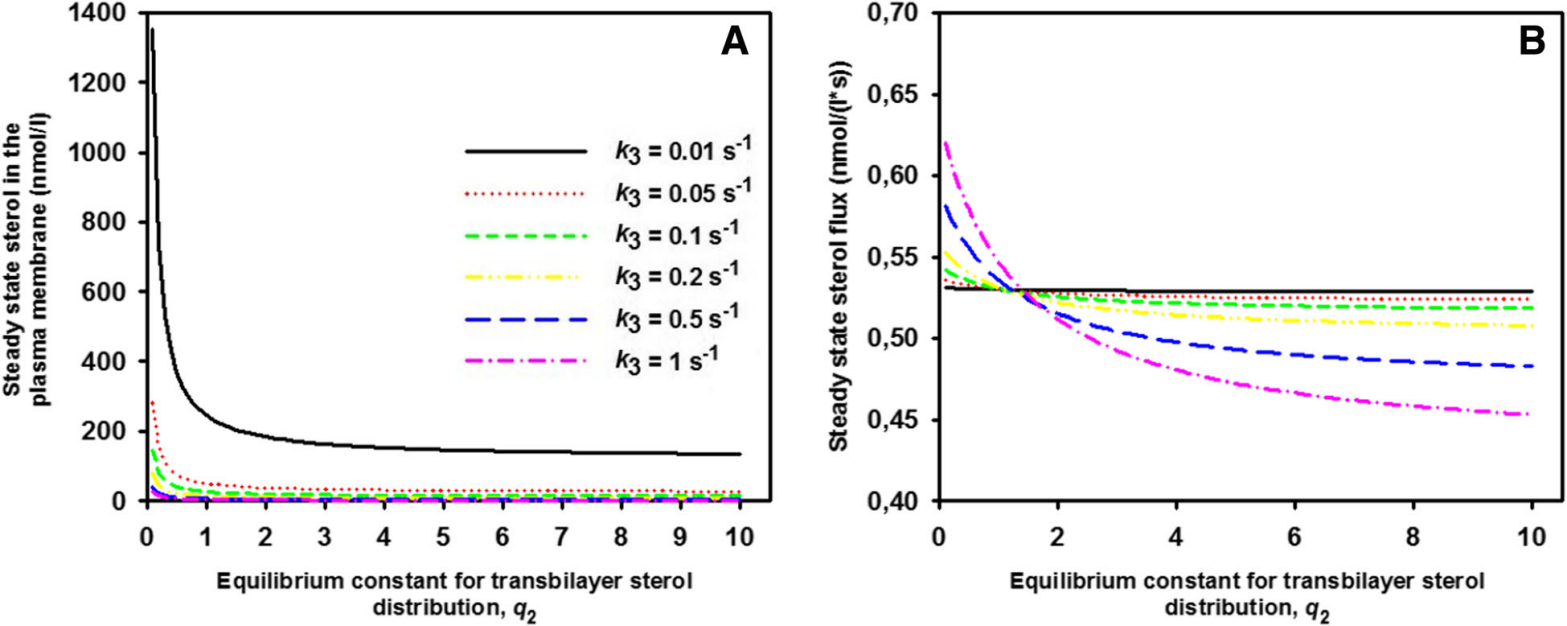
Sterol abundance in the PM and steady state sterol flux across the PM decrease non-linearly with increasing *q*_2_. Steady state sterol abundance in the PM (**a** *S*_T_, sum of Eq. 14a, 14b) and steady state sterol flux across the PM (**b** Eq. 16) as function of the passive sterol distribution between both PM leaflets (*q*_2_) for the indicated rate constants of intracellular sterol release (*k*_3_). Other parameters were *v*_1_ = 1 mol/s and *k*_− 2_ = 1 s^− 1^, respectively. See text for further explanations

Here, *q*_2_ is the equilibrium constant of the sterol flip-flop process, i.e., ${q}_2=\frac{k_2}{k_{-2}}=\frac{S_2^{eq}}{S_1^{eq}}$, where ${S}_1^{eq}$ and ${S}_2^{eq}$ are the sterol concentration at equilibrium in the outer and inner leaflet, respectively. Thus, we see that the steady state ratio of sterol between inner and outer leaflet, *Q*, is independent of the steady state sterol influx, *v*_1_. In fact, *Q* is described by a ratio of rate constants making that it has no units. Accordingly, the absolute values of kinetic rate constants are not relevant in making predictions about the steady state sterol ratio in the PM, only their relationship to each other. This is important as such values are not known for membranes in living cells. We see that *Q* is always smaller than the equilibrium ratio, i.e., as long as *k*_3_, the rate constant describing sterol exit from the PM is larger than zero. But how much does cytoplasmic sterol release affect this ratio or is the effect negligible under physiological settings? In case of *k*_3_ = 0, one finds again the equilibrium sterol distribution across the PM (i.e., in this case *Q* = *q*_2_). In Fig. 2a, the relationship between the equilibrium constant for passive sterol transbilayer distribution, *q*_2_, is plotted versus the steady state ratio, *Q* for varying release rate constants of sterol from the inner leaflet. For this calculation, the rate constant for sterol migration from the inner to the outer PM leaflet (sterol flopping) was set to *k*_− 2_ = 0.1 s^− 1^, while that in the opposite direction (sterol flipping) was varied between *k*_2_ = 0.01–1.0 s^− 1^, corresponding to an equilibrium ratio of *q*_2_ = 0.1–10. Sterol release from the cytoplasmic leaflet was also varied over a 100fold range from *k*_3_ = 0.01–1.0 s^− 1^. One can see that for *k*_3_ = 0.01 s^− 1^, which is 10fold smaller than the flop rate constant *k*_− 2_, the attainable steady state ratio of sterol in the two PM leaflets closely resembles the equilibrium distribution (Fig. 2a, black curve). In contrast, already when *k*_3_ = 0.05 s^− 1^ equal to half the rate constant for sterol flopping, *Q* deviates significantly from *q*_2_, and this difference grows as *k*_3_ is increased. To illustrate this further, we plotted the percentage of sterol in the inner leaflet for the same parameter values (Fig. 2b). One finds that the experimentally measured ~ 79% sterol in the inner leaflet of *hem1Δ* cells at steady state [31] requires *q*_2_ = 4.2 for a release rate constant of *k*_3_ = 0.01 s^− 1^corresponding to a passive equilibrium distribution of 80.77% sterol in the inner leaflet. Thus, the percentage of sterol in the inner leaflet at steady state (79%) is almost identical to the passive sterol distribution at thermodynamic equilibrium (≈81%) for this parameter combination, and such small differences cannot be detected in experiments [31]. In contrast, for a 10fold higher release rate constant of *k*_3_ = 0.1 s^− 1^, to obtain 79% sterol in the inner leaf of the PM at steady state requires *q*_2_ = 7.5, i.e. a passive equilibrium distribution of 88.23% sterol in the inner leaflet. At the same time, such increased sterol release on the cytosolic side would lower the total amount of sterol in the PM by about 10 fold (Fig. 3a). This is a rather drastic change, which has not been observed in experiments [31]. Thus, one might wonder under which physiological conditions the steady state sterol distribution in the PM would differ significantly from the equilibrium distribution. In other words, does the in vivo situation more resemble a state in which flip-flop is much faster than cytoplasmic sterol release and esterification in the ER? In this case, one would always have *k*_3_ < < *k*_2_, *k*_*−* 2_ and expect that *Q* approaches the equilibrium sterol transbilayer distribution. This is further analyzed in the thermodynamic analysis below.

#### Irreversible sterol import via Aus1/Pdr11 – thermodynamic analysis

To assess the extent by which continuous ergosterol import into yeast causes an out-of-equilibrium sterol distribution in the PM, we explored how the kinetic parameters affect the thermodynamic driving force for sterol influx. We define first the chemical potential difference of sterol between both leaflets under standard conditions in equilibrium

12 $$ \Delta {\mu}^0={\mu^0}_2-{\mu^0}_1= RT\cdot \ln \left(\frac{1}{q_2}\right) $$

It has been suggested that the chemical potential of sterol in both leaflets at steady state is not the same, and that especially the higher content of phosphatidylethanolamine could ‘draw’ sterol to the inner leaflet, establishing that *q*_2_ = *k*_2_/*k*_−2_ > 1 [45]. In addition, there is a non-equilibrium contribution to the asymmetric sterol distribution in the PM and for that, we define the chemical potential difference at steady state as:

13 $$ \Delta \mu =\Delta G={\mu}_2-{\mu}_1=\Delta {\mu}^0+ RT\cdot \ln \left(\frac{{\overline{S}}_2}{{\overline{S}}_1}\right)=- RT\cdot \ln \left(\frac{{\overline{v}}_2^{+}}{{\overline{v}}_2^{-}}\right)= RT\cdot \ln \left(\frac{{\overline{v}}_2^{-}}{{\overline{v}}_2^{+}}\right) $$

Here, the steady state forward (flip) and reverse rate (flop) for the transbilayer migration of sterol is defined as ${\overline{v}}_2^{+}={k}_2\cdot {\overline{S}}_1$ and ${\overline{v}}_2^{-}={k}_{-2}\cdot {\overline{S}}_2$, where we have $J={v}_1={k}_1\cdot {S}_0={\overline{v}}_2^{+}-{\overline{v}}_2^{-}$, for the low-substrate regime and $J={v}_1={v}_{\mathrm{max}}={\overline{v}}_2^{+}-{\overline{v}}_2^{-}$ for the high-substrate regime, which is easily verified using Eq. 3a, 3b. Using ${S}_T={\overline{S}}_1+{\overline{S}}_2$, we can rewrite Eq. 3a, 3b as:

14a $$ \overline{S_1}=\frac{k_{-2}\cdot {S}_T+J}{k_2+{k}_{-2}} $$

14b $$ \overline{S_2}=\frac{k_2\cdot {S}_T-J}{k_2+{k}_{-2}} $$

With that, the chemical potential difference for sterol between both leaflets at steady state (Eq. 13) reads:

15 $$ \Delta \mu =- RT\cdot \ln \left(\frac{\left({k}_{-2}\cdot {S}_T+{v}_1\right)}{\left({k}_2\cdot {S}_T-{v}_1\right)}\cdot {q}_2\right)=\Delta G= RT\cdot \ln \left(\frac{S_1^{eq}}{S_2^{eq}}\cdot \frac{\overline{S_2}}{\overline{S_1}}\right)= RT\cdot \ln \left(\frac{Q}{q_2}\right) $$

The expression in the large brackets in the right-hand term of Eq. 15 can be seen as a form of the mass action ratio for a non-equilibrium system (i.e. *Q*/*q*_2_). With that, we can express the steady state flux as function of the chemical potential difference of sterol between the PM leaflets [46]:

16 $$ J=\frac{k_2\cdot {S}_T\cdot \left(\exp \left(-\Delta \mu / RT\right)-1\right)}{q_2+\exp \left(-\Delta \mu / RT\right)} $$

This thermodynamic definition of the steady state flux is valid for linear import kinetics. In that case, sterol influx necessary to counterbalance a non-equilibrium difference in sterol abundance between both leaflets is a monotonically decreasing non-linear function of the equilibrium sterol distribution between both leaflets (Fig. 3b). That is, the higher the passive sterol distribution towards the inner PM leaflet, the lower is the steady state sterol flux into the cell. In other words, the faster sterols flip spontaneously across the PM compared to cytoplasmic sterol release the closer is the system to thermodynamic equilibrium. Vice versa, higher sterol chemical potential differences due to large sterol release from the inner leaflet and esterification in the ER, as described by rate constant *k*_3_, result in larger steady state fluxes (e.g. compare pink dot-dashed curve with *k*_3_ = 1 s^− 1^ and green dashed curve with *k*_3_ = 0.1 s^− 1^ in Fig. 3b). The rate constant *k*_3_ in the model of Eq. 1 comprises cytoplasmic sterol release and its esterification in the ER. Consequently, controlling the ‘sterol sink’ in the ER, i.e. sterol esterification by Are2 should directly relate to the magnitude of out-of-equilibrium sterol flux across the PM. Positive values of Δμ would give a negative flux, i.e., a flux reversion from the inner towards the outer leaflet. However, such a situation cannot happen, as long as we have *k*_3_ ≥ 0. This can be easily seen, when using Eq. 11, in Eq. 15 to get:

17 $$ \Delta G=\Delta \mu = RT\cdot \ln \left(\frac{Q}{q_2}\right)= RT\cdot \ln \left(\frac{1}{1+{k}_3/{k}_{-2}}\right) $$

For *k*_3_ = 0, we get Δ*G* = 0, which is the thermodynamic equilibrium. In that situation we have ${\overline{v}}_2^{+}={\overline{v}}_2^{-}$, meaning that no net flux across the PM would remain. So, how different is the ergosterol distribution between the two PM leaflets at a physiologically reasonable steady state from its equilibrium value? Again, the total number of ergosterol molecules in the PM is *S*_T_ = 7∙10^7^ per yeast cell. Even though this is widely speculative, let’s assume that passive sterol flip and flop have a half time of t_1/2_ = 1.73 s (*k*_2_ = 0.4 s^− 1^) and t_1/2_ = 6.93 s (*k*_*−* 2_ = 0.1 s^− 1^), respectively. This corresponds to a passive distribution of sterol between the outer and inner PM leaflet of q_2_ = 4 and thereby to 20% of ergosterol (1.4∙10^7^ molecules) in the outer and 80% (5.6∙10^7^ molecules) in the inner PM leaflet at thermodynamic equilibrium (i.e. in the absence of any intracellular sterol release from the PM described by rate constant *k*_3_). In the presence of a cytoplasmic release from the PM with rate constant of *k*_3_ = 0.01 s^− 1^ (t_1/2_ = 69 s), 7∙10^5^ ergosterol molecules would leave the PM per second. With that, we have according to Eqs. 15 and 16 at steady state Q= $\frac{\overline{S_2}}{\overline{S_1}}$ =3.64, thus, 21.56% of ergosterol (1.51∙10^7^ molecules) in the outer and 78.44% (5.49∙10^7^ molecules) in the inner leaflet at steady state. This is only slightly different from the equilibrium situation, above. Such a difference is too small to be detectable in a steady state quenching experiment of DHE in the yeast PM, for example [31]. Supporting this notion, we found that energy poisoning of cells did not affect the measured distribution of DHE across the yeast PM [31]. For these values, we get for the Gibbs free energy:

18 $$ \Delta G= RT\cdot \ln \left(\frac{1}{1+{k}_3/{k}_{-2}}\right)= RT\cdot \ln \left(\frac{1}{1+0.01{s}^{-1}/0.1{s}^{-1}}\right)=-0.24 kJ/ mol $$

This value of ΔG = − 0.24 kJ/mol is a rather low thermodynamic driving force, and the system will remain close to thermodynamic equilibrium. Let’s increase the rate constant for sterol release from the inner leaflet, *k*_3_, 5fold, i.e., from *k*_3_ = 0.01 s^− 1^ to *k*_3_ = 0.05 s^− 1^ while keeping *q*_2_ = 4.0 with a sterol flip and flop rate constant of *k*_2_ = 0.4 s^− 1^ and *k*_*−* 2_ = 0.1 s^− 1^, respectively. This corresponds to ΔG = − 1.03 kJ/mol. Again, this value for the Gibbs free energy change would also be obtained for very different values of the rate constants, as long as their ratio is the same. For example, one might have in a yeast cell, *k*_2_ = 0.04 s^− 1^ (t_1/2_ = 17.3 s) and *k*_*−* 2_ = 0.01 s^− 1^ (t_1/2_ = 69.3 s). In that case, the above example calculations stay valid for *k*_3_ = 0.001 s^− 1^ (t_1/2_ = 693.2 s) to *k*_3_ = 0.005 s^− 1^ (t_1/2_ = 138.6 s). This is important, as the absolute values for sterol flip-flop, for example, have been debated and might vary over a wide range depending on lipid composition [47].

In parallel, we get using Eqs. 11 and 17; Q= $\frac{\overline{S_2}}{\overline{S_1}}$ =0.4 s^− 1^/(0.1 s^− 1^ + 0.05 s^− 1^) = 0.04 s^− 1^/(0.01 s^− 1^ + 0.005 s^− 1^) = 2.67 (i.e. 72.75% sterol in the inner leaflet). Such a change in sterol asymmetry might be detectable by DHE quenching experiments. However, at the same time the total amount of sterol in the PM would drop to less than 20% of what it was at *k*_3_ = 0.01 s^− 1^ (for *k*_2_ = 0.1 s^− 1^) and at *k*_3_ = 0.001 s^− 1^ (for *k*_2_ = 0.01 s^− 1^) This, again, has not been observed in experiments, at least not in the absence of de novo ergosterol synthesis [27, 28, 31]. Thus, we can conclude that cytoplasmic release of ergosterol is likely slow compared to sterol flip-flop rates (i.e. *k*_*−* 2_ ≥ 10∙ *k*_3_) causing only moderate deviations from the equilibrium sterol distribution in the PM.

For such small deviations from equilibrium, we can linearize the flux-force relationship of Eq. 16 and get [46]:

19 $$ J=-\Delta \mu \cdot \frac{S_T}{RT}\cdot \left(\frac{k_2\cdot {k}_{-2}}{k_2+{k}_{-2}}\right)=-\Delta \mu \cdot \frac{S_T\cdot {k}_2}{RT\left({q}_2+1\right)} $$

This resembles the Onsager flux force relationship for linear irreversible thermodynamics; *J* = *L*·*X*, with the steady state flux being *J* = *v*_1_, *X* being the driving force (here X = -Δμ) and *L* being the transport coefficient (or ‘conductance’ (equivalent to a diffusion constant), here:

20 $$ L=\frac{S_T\cdot {k}_2}{RT\left({q}_2+1\right)}=\frac{S_T\cdot {k}_2\cdot {k}_{-2}}{RT\cdot \tau } $$

With *S*_*T*_ = [*v*_1_ ∙ (*τ*^−1^ + *k*_3_)]/[*k*_2_ ∙ *k*_3_]) the ‘conductance’ is determined by a combination of all rate constants of sterol transport across the PM relative to the Boltzmann factor (the higher the flip-flop rates, the faster is sterol exchange; where *τ* = (*k*_2_ + *k*_− 2_)^−1^ is the fluctuation relaxation time). From that, we can derive the heat loss due to entropy production, σ, per unit membrane area as [46]:

21 $$ T\cdot \sigma =J\cdot X=-\Delta \mu \cdot {v}_1={\left(\Delta \mu \right)}^2\cdot \frac{S_T\cdot {k}_2\cdot {k}_{-2}}{RT\cdot \tau } $$

Thus, the larger the sterol flux across the PM, the higher is the entropy production, as expected for a non-equilibrium steady state. Again, based on the above quantitative arguments, we expect the flux to be small causing only small deviations from thermodynamic equilibrium distributions. The directionality of this steady state influx is ensured by Aus1/Pdr11 transporters constantly hydrolyzing ATP. Can we include this in our analysis?

#### Reversible sterol import via Aus1/Pdr11 – steady state analysis

It is generally believed that ABC transporters shuttle their substrate in an unidirectional manner driven by ATP hydrolysis. However, some of those transporters like the LmrA multidrug transporter of *Lactococcus lactis* can act also in the opposite direction under ATP depletion condition and reversed substrate gradients [48]. While the simultaneous existence of a reverse sterol and ATP gradient to synthesize ATP by Aus1/Pdr11 in yeast is rather implausible, the possibility exists that the ABC transporters Aus1/Pdr11 could carry out a reversible transport cycle. In this scenario, the ABC transporters could pump sterols also out of the cell, but the excess of ATP compared to ADP and phosphate on the substrate-entry site would ensure the directionality of sterol flux in the import direction [46]. Such a mechanism has been proposed for the ABC transporter associated with antigen processing (TAP) [49]. In that case, we have for our system:

22 $$ {S}_0\overset{v_1}{\leftrightarrow }{S}_1\overset{v_2}{\leftrightarrow }{S}_2\overset{v_3}{\to } $$

We consider sterol in the outer PM leaflet (*S*_1_) and the inner PM leaflet (*S*_2_) with constant concentration of ATP, ADP and phosphate, i.e. A:=[ATP] and P:=[ADP]·[PO_4_^2−^] and the rates ${v}_1={k}_1^{\ast}\cdot {S}_0-{k}_{-1}^{\ast}\cdot {S}_1$, *v*_2_ = *k*_2_ ⋅ *S*_1_ − *k*_−2_ ⋅ *S*_2_ and *v*_3_ = *k*_3_ ⋅ *S*_2_ resulting in pseudo-first order rate constants for the import as ${k}_1^{\ast }={k}_1\cdot A$ and ${k}_{-1}^{\ast }={k}_{-1}\cdot P$. This gives the ODE system:

23a, b $$ {\displaystyle \begin{array}{l}\frac{dS_1}{dt}={v}_1-{v}_2={k}_1^{\ast}\cdot {S}_0-\left({k}_{-1}^{\ast }+{k}_2\right)\cdot {S}_1+{k}_{-2}\cdot {S}_2\\ {}\frac{dS_2}{dt}={v}_2-{v}_3={k}_2\cdot {S}_1-\left({k}_{-2}+{k}_3\right)\cdot {S}_2\end{array}} $$

One can again show, that only one steady state flux exists, which is *v*_1_ = *v*_2_ = *v*_3_ = *J*. Thus, sterol fluxes due to i) Aus1/Pdr11-mediated sterol influx (*v*_1_), ii) sterol flip-flop across the PM (*v*_2_), and iii) due sterol release from the PM followed by sterol esterification in the ER (*v*_3_) become equal at steady state. The steady state amount of sterol in the outer and inner leaflet of the PM reads:

24a $$ \overline{S_1}=\frac{k_1\cdot {S}_0\cdot \left({k}_{-2}+{k}_3\right)}{k_{-1}^{\ast}\cdot {k}_{-2}+\left({k}_{-1}^{\ast }+{k}_2\right)\cdot {k}_3} $$

24b $$ \overline{S_2}=\frac{k_1\cdot {k}_2\cdot {S}_0}{k_{-1}^{\ast}\cdot {k}_{-2}+\left({k}_{-1}^{\ast }+{k}_2\right)\cdot {k}_3} $$

These expressions can be adapted for the high-substrate regime (b)) by replacing *v*_1_ = *k*_1_∙*S*_0_ with *v*_1_ = *v*_max_. Importantly, the steady state ratio of sterol between the two PM leaflets is again independent of the activity or abundance of Aus1/Pdr11, as it reads:

25 $$ \overline{S_2}/\overline{S_1}=\left(\frac{k_1\cdot {k}_2\cdot {S}_0}{k_{-1}^{\ast}\cdot {k}_{-2}+\left({k}_{-1}^{\ast }+{k}_2\right)\cdot {k}_3}\right)\cdot {\left(\frac{k_1\cdot {S}_0\cdot \left({k}_{-2}+{k}_3\right)}{k_{-1}^{\ast}\cdot {k}_{-2}+\left({k}_{-1}^{\ast }+{k}_2\right)\cdot {k}_3}\right)}^{-1}=\frac{k_2}{k_{-2}+{k}_3}=Q $$

The most right side of Eq. 25 is identical to that of Eq. 11, showing that assuming reversibility of the sterol import step does not affect the steady state sterol distribution in the PM. This is an important result with the consequence that the whole thermodynamic flux analysis presented above remains valid also for reversible and ATP-dependent sterol import by Aus1/Pdr11. In contrast to the irreversible sterol import (Eq. 1), flux control is now shared between the forward and backward step in the first transport step (not shown). Note that the relations above, i.e. expressions relating the flux *J* (i.e., *v*_1_) to the chemical potential differences, those quantifying sterol abundance in each leaflet at steady state (Eqs. 3a, 3b, 11, 17 and 24a, 24b) and the steady state transbilayer sterol distribution in the PM (Eq. 25) are generally valid for linear import kinetics. In the following, we will only use *v*_1_ for the Aus1/Pdr11-catalyzed sterol import step, irrespective of *v*_1_ = *k*_1_ ⋅ *S*_0_ (low-substrate regime, a), above) or *v*_1_ = *v*_max_ (high-substrate regime, b), above).

### Model of sterol transport in yeast with two sterol pools in each PM leaflet

#### Linearized model of sterol complex formation in the PM only

The model analysis so far predicts that passive sterol redistribution to the inner leaflet (i.e., preferred inward flipping, q_2_ > 0) is the main driving force for the observed sterol asymmetry in the PM with little counterbalance from active transport. In the following, a mechanism is discussed by which sterol can be enriched in one leaflet without invoking differing flip and flop rates for passive sterol exchange between the leaflets. It has been suggested that the PM of eukaryotic cells is sub-compartmentalized into an active sterol pool, which rapidly responds to changes in lipid composition or sterol abundance, and a ‘passive’ pool, which is restraint to the PM by complex formation with phospholipids [15, 50]. The molecular mechanisms of sterol sequestration in the complexed ‘inactive’ pool are not clear at the moment, but could involve preferred interaction with some lipid species, like sphingolipids, or with membrane-embedded or –associated proteins [14, 50, 51]. The ‘active’ pool in each leaflet is meant as being freely available for exchange including complex formation and flip-flop to the opposite leaflet. Its definition is routed in a thorough thermodynamic analysis of phospholipid-cholesterol phase diagrams in lipid model systems, in which a steep increase in cholesterol’s chemical activity, *a*, with *a* = exp(*μ*/*k*_b_∙*T*) at critical cholesterol mole fractions was found [15, 52]. The chemical activity surmises all molecular interactions of sterol in the bilayer defining its available volume as function of sterol concentration [52, 53]. Due to their concentration-dependent lipid condensing effects, higher sterols like cholesterol ergosterol impact the available volume in the bilayer which in turn affects sterol-phospholipid interactions. Several physico-chemical models have been put forward to explain the highly non-linear dependence of cholesterol’s chemical potential in membranes on bilayer sterol mole fraction, as recently reviewed [8]. To keep our analysis simple and transparent, we employ a model based on mass-action kinetics involving sterol-phospholipid complexes [15, 52, 53]. This approach also has another advantage, namely that the sterol binding partner potentially can be other types of molecules, like membrane proteins. To include a complexed sterol pool in our analysis, the irreversible import model was extended to.

26a $$ {S}_0\overset{v_1}{\to }{S}_1\overset{v_2}{\leftrightarrow }{S}_2\overset{v_3}{\to } $$

26b, c $$ {\displaystyle \begin{array}{l}{nS}_1+{mP}_1\overset{v_4}{\leftrightarrow }{C}_1\\ {}{nS}_2+{mP}_2\overset{v_5}{\leftrightarrow }{C}_2\end{array}} $$

Now, we define the active or free sterol in the outer PM leaflet as *S*_1_ and the active sterol in the inner PM leaflet as *S*_2_. In both leaflets, *n* molecules of free sterol can bind to *m* molecules of phospholipids, designated as *P*_1_ for the outer and *P*_2_ for the inner leaflet, respectively. Such binding results in formation of complexes named *C*_1_ for the outer and *C*_2_ for the inner leaflet, respectively. This is justified by the observation, that condensed complexes can form between sterols and sphingolipids, as primarily found in the outer PM leaflet, but also with PS, being enriched in the cytoplasmic PM leaflet [15, 52, 54]. Condensed complexes are supposed to cover less membrane area than the sum of the contributing constituents would in the non-complexed state, thereby accounting for cholesterol’s condensing effect on lipid membranes [53, 55]. The free or active sterol can move between the leaflets and across the bilayer with rate *v*_2_ in our model, which ensures that free and complexed pools in each leaflet are kinetically coupled. That is, free sterol in the outer leaflet, *S*_1_, flipped to the inner can be replenished by dissociation of the condensed complex in the outer leaflet (with rate *v*_4_). Similarly, active sterol released into the cytoplasm from the inner leaflet (with rate *v*_3_) can be replenished from both complexed sterol pools; from dissociation of the condensed complex in the inner leaflet (with rate *v*_5_) and from dissociation of the condensed complex in the outer leaflet (with rate *v*_4_) followed by flip of the liberated free sterol from the outer to the inner leaflet (with rate *v*_2_). For simplicity, we consider only 1st order complex formation setting *n* = *m* = 1 as previously suggested for ternary lipid mixtures in model membrane vesicles [56]. Further, we assume for the moment the phospholipids to be in excess for the binding such that we obtain pseudo first order rate constants for binding in the outer leaflet, *k*_4_ = *k*_4_*∙ *P*_1_·and in the inner leaflet, *k*_5_ = *k*_5_*∙ *P*_2_. This implies that sterol inserted into the PM from extracellular sources to be a minor component, such that complex formation does not consume the sterol binding partners. This constrain is now set for simplicity and transparency of the analysis but will later be removed (see below). Together, this gives the following transport rates: *v*_1_ = const., *v*_2_ = *k*_2_ ⋅ *S*_1_ − *k*_−2_ ⋅ *S*_2_, *v*_3_ = *k*_3_ ⋅ *S*_2_, *v*_4_ = *k*_4_ ⋅ *S*_1_ − *k*_−4_ ⋅ *C*_1_ and *v*_5_ = *k*_5_ ⋅ *S*_2_ − *k*_−5_ ⋅ *C*_2_. The resulting ODE system reads:

27a-d $$ {\displaystyle \begin{array}{l}\frac{dS_1}{dt}={v}_1-{v}_2-{v}_4={k}_1\cdot {S}_0-\left({k}_2+{k}_4\right)\cdot {S}_1+{k}_{-2}\cdot {S}_2+{k}_{-4}\cdot {C}_1\\ {}\frac{dS_2}{dt}={v}_2-{v}_3-{v}_5={k}_2\cdot {S}_1-\left({k}_{-2}+{k}_3+{k}_5\right)\cdot {S}_2+{k}_{-5}\cdot {C}_2\\ {}\frac{dC_1}{dt}={v}_4={k}_4\cdot {S}_1-{k}_{-4}\cdot {C}_1\\ {}\frac{dC_2}{dt}={v}_5={k}_5\cdot {S}_2-{k}_{-5}\cdot {C}_2\end{array}} $$

The steady state amount of active sterol in the outer and inner leaflet of the PM is:

28a $$ \overline{S_1}=\frac{k_1\cdot {S}_0\cdot \left({k}_{-2}+{k}_3\right)}{k_2\cdot {k}_3}=\frac{v_1\cdot \left({k}_{-2}+{k}_3\right)}{k_2\cdot {k}_3} $$

28b $$ \overline{S_2}=\frac{k_1\cdot {k}_2\cdot {S}_0}{k_2\cdot {k}_3}=\frac{k_1\cdot {S}_0}{k_3}=\frac{v_1}{k_3} $$

This is identical to the one-pool irreversible model (Eq. 3a and 3b), showing that active sterol moves freely between the two PM leaflets. That does not mean, though, that the complexed sterol pools in each leaflet are independent of each other. In fact, it can be easily seen from the steady state solutions for the sterol in complexes, that:

29a $$ \overline{C_1}=\frac{k_4\cdot {v}_1\cdot \left({k}_{-2}+{k}_3\right)}{k_2\cdot {k}_3\cdot {k}_{-4}}=\frac{q_4\cdot {v}_1\cdot \left({k}_{-2}+{k}_3\right)}{k_2\cdot {k}_3}={q}_4\cdot \overline{S_1} $$

29b $$ \overline{C_2}=\frac{k_5\cdot {v}_1}{k_{-5}\cdot {k}_3}=\frac{q_5\cdot {v}_1}{k_3}={q}_5\cdot \overline{S_2} $$

Here, the equilibrium constants for complex formation in the outer and inner leaflet are *q*_4_ = *k*_4_/*k*_−4_ and *q*_5_ = *k*_5_/*k*_−5_, respectively. Using Eqs. 28a and 28b in 29a, one finds that sterol complex in the outer leaflet at steady state can be expressed as function of free sterol in the inner leaflet as

29c $$ \overline{C_1}=\frac{q_4\cdot \overline{S_2}\cdot \left({k}_{-2}+{k}_3\right)}{k_2} $$

Similar relations can be found to express sterol complex in the inner leaflet at steady state as function of free sterol in the outer leaflet (not shown), clearly establishing that all four sterol pools in the PM are connected with each other. The total amount of sterol in the outer leaflet, *S*_T_^o^, and in the inner leaflet, *S*_T_^i^, becomes:

30a, b $$ {\displaystyle \begin{array}{l}{S}_T^o=\overline{S_1}+\overline{C_1}=\left(1+{q}_4\right)\cdot \overline{S_1}\\ {}{S}_T^i=\overline{S_2}+\overline{C_2}=\left(1+{q}_5\right)\cdot \overline{S_2}\end{array}} $$

The steady state ratio of sterol between the two PM leaflets reads:

31 $$ {S}_T^i/{S}_T^o=\left(\overline{S_2}+\overline{C_2}\right)/\left(\overline{S_1}+\overline{C_1}\right)=\frac{\left(1+{q}_5\right)\cdot \overline{S_2}}{\left(1+{q}_4\right)\cdot \overline{S_1}}=\frac{k_2\cdot {k}_{-4}\cdot \left({k}_5+{k}_{-5}\right)}{\left({k}_{-2}+{k}_3\right)\cdot \left({k}_4+{k}_{-4}\right)\cdot {k}_{-5}} $$

This can be simplified to:

32 $$ {S}_T^i/{S}_T^o=\frac{k_2}{k_{-2}+{k}_3}\cdot \frac{\left(1+{q}_5\right)}{\left(1+{q}_4\right)}=Q\cdot \frac{\left(1+{q}_5\right)}{\left(1+{q}_4\right)} $$

**Fig. 4:**
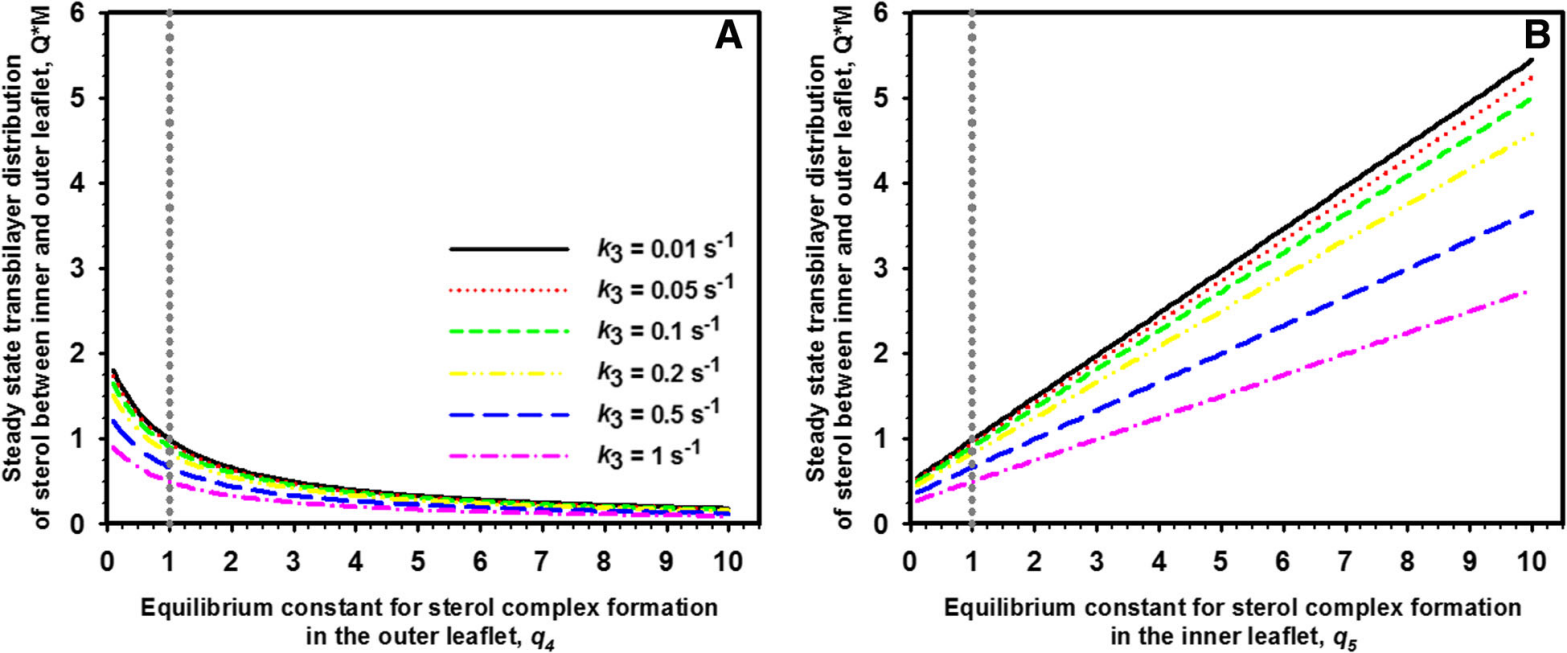
Preferred complex formation of sterol in the inner leaflet counteracts increasing sterol release rates in attaining sterol asymmetry in the PM. Steady state transbilayer sterol distribution (ratio Q∙M, Eq. 32) as function of equilibrium constant for sterol complex formation in the outer leaflet (**a** varying *q*_4_) or in the inner leaflet of the PM (**b** varying *q*_5_). The vertical dotted grey line indicates the equilibrium constant for the complex formation in the corresponding other leaflet (i.e., *q*_5_ = 1 in **a**; *q*_4_ = 1 in **b**). While *k*_3_ was varied as shown in panel (**a**), flip-flop rate constants were set to *k*_2_ = *k*_− 2_ = 1 s^− 1^, respectively. A ratio Q∙M = 1 corresponds to a symmetric sterol distribution between both leaflets, while Q∙M > 1; Q∙M < 1 means sterol enrichment in the inner and outer leaflet, respectively

From Eq. 31/32, one can draw two import conclusions; first, the steady state transbilayer sterol distribution is also for the two-pool model independent of sterol influx, *v*_1_. Second, introducing two sterol pools (free and in complex) into each PM leaflet modifies the original steady state transbilayer sterol distribution, Q, by the factor $M=\frac{\left(1+{q}_5\right)}{\left(1+{q}_4\right)}$. The steady state ratio of sterol between the two PM leaflets as function of the equilibrium constants for complex formation in either leaflet is shown Fig. 4. Here, the equilibrium constant not being varied is set equal to 1 (i.e. *q*_5_ = 1 in Fig. 4a and *q*_4_ = 1 in Fig. 4b; dotted grey line), meaning that half of total leaflet sterol is in complexes and half is free in either case. The equilibrium constant for passive sterol flip-flop, *q*_2_, is also set to one, meaning that active sterol does not show any preference for either leaflet in this model. More sterol in complexes in the outer leaflet, i.e., less being free or ‘active’ lowers the total amount of sterol in the inner side of the PM in a non-linear manner (Fig. 4a). If complex formation takes place primarily in the inner leaflet, the steady state ratio of sterol is shifted to this side of the bilayer (Fig. 4b). Thus, despite identical passive flip-flop rates, sterol asymmetry can be controlled in this model by differing interaction of sterol with phospholipids in either leaflet. As before for the one-pool model, increasing non-vesicular sterol outflux described by rate constant *k*_3_ lowers the attainable steady state sterol ratio between both PM leaflets. One also sees that sterol enrichment in the inner leaflet on expense of the outer is only possible if complex formation takes place preferentially with phospholipids in the inner PM leaflet (i.e., we must have *q*_5_ > > *q*_4_). Interestingly ergosterol seems to be excluded from gel-like domains formed by outer-leaflet sphingolipids in the yeast PM [57]. This could effectively lower complex formation in the outer PM leaflet (i.e., *q*_4_). In addition, sterols might be drawn to the inner half of the PM to reduce spontaneous bilayer curvature caused by the inverted cone shape of PE species and by charge repulsion of PS in the inner leaflet [45, 58]. Both processes could transiently immobilize some sterol in complexes, thereby raising *q*_5_ and stabilizing sterol asymmetry in the yeast PM. Alternatively, ergosterol might not at all be in complex with phospholipids in the inner leaflet but rather bind to cytoplasmic proteins accessing sterol from the inner PM leaflet. In fact, peripheral proteins can sequester sterols in the PM. This has been shown for perifringolysin derivatives bound to the outer PM leaflet which prevented non-vesicular sterol transport to the ER [59, 60]. Accordingly, in our model Eq. 26b, c could also describe formation of protein –sterol complexes which would enrich ergosterol in the inner compared to the outer PM leaflet. Clearly more work is required to determine the underlying molecular mechanisms of sterol sequestration.

For the 2-pool model, the steady state sterol fluxes across the PM read:

33 $$ {\displaystyle \begin{array}{l}\overline{v_2}=\overline{v_3}={v}_1\\ {}\overline{v_4}=\overline{v_5}=0\end{array}} $$

That is, like in the irreversible one-pool model, only one flux exists at steady state, which is entirely set by the sterol import rate via Aus1/Pdr11, irrespective of whether the system is in the low- or high-substrate regime. Sterol in complexes with leaflet-specific phospholipids or with membrane-attached or –embedded proteins does not contribute to the steady state sterol flux in this model. This is an important conclusion, as it shows that sterol flux between various membrane pools can be fast, even though sterol gradients are stable at steady state.

Finally, we compare the expressions for the total PM associated sterol in case of the irreversible one- and two pool models, respectively. For the one-pool model, we had (see above and Fig. 3a):

34 $$ {S}_T={\overline{S}}_1+{\overline{S}}_2=\frac{v_1\cdot \left({k}_2+{k}_{-2}+{k}_3\right)}{k_2\cdot {k}_3} $$

With Eqs. 28a, 28b and 30a, b, we get for the two-pool model

35 $$ {S}_T=\overline{S_1}+\overline{C_1}+\overline{S_2}+\overline{C_2}=\frac{v_1\cdot \left({k}_2\cdot \left(1+{q}_5\right)+\left({k}_{-2}+{k}_3\right)\cdot \left(1+{q}_4\right)\right)}{k_2\cdot {k}_3} $$

By comparing Eqs. 34 and 35, one sees, that the amount of sterol in the PM at steady state will be a linear increasing function for increasing *q*_4_ or *q*_5_. That is, complex formation in either leaflet retains sterol in the PM. For *q*_4_ and *q*_5_ being zero, we recover the solution for the one pool model, Eq. 34.

### Steady state model including two sterol pools in each PM leaflet, transport to the ER and esterification

#### Linearized model of sterol complex formation in the PM and ER

Cholesterol in mammalian cells and ergosterol in yeast are very abundant in the PM compared to the ER, even though the biochemical machinery for sterol synthesis and esterification reside mostly in the ER, and there is continuous sterol exchange taking place between both compartments [4, 10, 13, 14]. It has been suggested that difference in lipid composition with strongly varying affinity for sterols exist between both organelles which might be responsible for establishment and maintenance of this strong sterol gradient between PM and ER [8, 13, 61]. Thermodynamic equilibrium models have been put forward which assume that sterols in the PM exist in a free or ‘active’ pool and in a pool bound with differing affinity to phospholipids in the PM versus the ER, which could result in identical chemical potentials for sterols in each membrane despite differing concentrations [10, 15, 62]. How a non-equilibrium steady state of sterol influx and esterification would affect such a 2-pool model of sterol in the PM and ER has not been studied. Here, we extend the two-pool model for the PM to include rapid non-vesicular sterol exchange with the ER followed by explicit consideration of sterol esterification in this compartment. The latter allows us to dissect separately the contribution of non-vesicular sterol exchange between PM and ER and sterol esterification. We make again use of the fact, that sterol extraction from membranes by STPs but not diffusion of protein-sterol complexes in the cytoplasm is rate-limiting for sterol exchange between PM and ER, justifying the use of an ODE system to describe the following process:

36a $$ {S}_0\overset{v_1}{\to }{S}_1\overset{v_2}{\leftrightarrow }{S}_2\overset{v_3}{\leftrightarrow }{S}_3\overset{v_7}{\to } $$

36b, c, d $$ {\displaystyle \begin{array}{l}{nS}_1+{mP}_1\overset{v_4}{\leftrightarrow }{C}_1\\ {}{nS}_2+{mP}_2\overset{v_5}{\leftrightarrow }{C}_2\\ {}{nS}_3+{mP}_3\overset{v_5}{\leftrightarrow }{C}_3\end{array}} $$

As before, we have the active or free sterol in the outer PM leaflet as *S*_1_ and the active sterol in the inner PM leaflet as *S*_2_. In both leaflets, *n* = 1 molecules of free sterol can bind to *m* = 1 molecules of phospholipids, designated as *P*_1_ for the outer and *P*_2_ for the inner leaflet, respectively. Such binding results in formation of complexes named *C*_1_ for the outer and *C*_2_ for the inner leaflet, respectively. Only the free or active sterol can move between the leaflets and across the bilayer. For simplicity, we consider again the phospholipids to be in excess for the binding such that we obtain pseudo first order rate constants for binding in the outer leaflet, *k*_4_ = *k*_4_*∙ P_1_·and in the inner leaflet, *k*_5_ = *k*_5_*∙ P_2_. Exactly the same approach is used to describe sterol complex formation with phospholipids of the ER in Eq. 36b, c, d, where *S*_3_ is the freely moving sterol pool, and *C*_3_ describes the sterol complexes in the ER. In addition, we consider now reversible sterol exchange between PM and ER and assume that forward and backward rate constants for sterol flip flop and for exchange between PM and ER are identical giving *q*_2_ = *q*_3_ = 1. Sterol esterification by Are2 is creating an irreversible sink of non-esterified sterol described by rate *v*_7_. It follows linear kinetics, which can be justified in the low substrate regime using a linearization of the Michaelis-Menten rate law, as described in Eqs. 5 and 6 for the Aus1/Pdr11 membrane transporter (see above). This gives the following transport rates: *v*_1_ = const., *v*_2_ = *k*_2_ ⋅ (*S*_1_ − *S*_2_), *v*_3_ = *k*_3_ ⋅ (*S*_2_ − *S*_3_), *v*_4_ = *k*_4_ ⋅ *S*_1_ − *k*_−4_ ⋅ *C*_1_, *v*_5_ = *k*_5_ ⋅ *S*_2_ − *k*_−5_ ⋅ *C*_2_, *v*_6_ = *k*_6_ ⋅ *S*_3_ − *k*_−6_ ⋅ *C*_3_ and *v*_7_ = *k*_7_ ⋅ *S*_3_ Thus, net sterol transfer from the outer to the inner PM leaflet and from the PM to the ER takes place as long as *v*_2_,*v*_3_ > 0, which is ensured as long as *S*_1_ > *S*_2_ > *S*_3_. The resulting ODE system reads:

37a-f $$ {\displaystyle \begin{array}{l}\frac{dS_1}{dt}={v}_1-{v}_2-{v}_4={k}_1\cdot {S}_0-\left({k}_2+{k}_4\right)\cdot {S}_1+{k}_2\cdot {S}_2+{k}_{-4}\cdot {C}_1\\ {}\frac{dS_2}{dt}={v}_2-{v}_3-{v}_5={k}_2\cdot {S}_1-\left({k}_2+{k}_3+{k}_5\right)\cdot {S}_2+{k}_{-5}\cdot {C}_2+{k}_3\cdot {S}_3\\ {}\frac{dS_3}{dt}={v}_3-{v}_6-{v}_7={k}_3\cdot {S}_2-\left({k}_3+{k}_6+{k}_7\right)\cdot {S}_3+{k}_{-6}\cdot {C}_3\\ {}\frac{dC_1}{dt}={v}_4={k}_4\cdot {S}_1-{k}_{-4}\cdot {C}_1\\ {}\frac{dC_2}{dt}={v}_5={k}_5\cdot {S}_2-{k}_{-5}\cdot {C}_2\\ {}\frac{dC_3}{dt}={v}_6={k}_6\cdot {S}_3-{k}_{-6}\cdot {C}_3\end{array}} $$

The steady state amount of active sterol in the outer and inner leaflet of the PM now reads:

38 $$ \overline{S_1}={v}_1\cdot \left(\frac{1}{k_2}+\frac{1}{k_3}+\frac{1}{k_7}\right) $$

39 $$ \overline{S_2}=\frac{v_1\cdot \left({k}_3+{k}_7\right)}{k_3\cdot {k}_7} $$

Setting $r=\frac{k_7}{k_3+{k}_7}$ as a weighted rate constant for sterol esterification, Eqs. 37a-f and 38 read

40 $$ \overline{S_1}=\frac{v_1\cdot \left({k}_3\cdot r+{k}_2\right)}{k_2\cdot {k}_3\cdot r} $$

41 $$ \overline{S_2}=\frac{v_1}{k_3\cdot r} $$

Thus, the expressions for the amount of free or ‘active’ sterol in the outer and inner PM leaflet at steady state contain now the weighted rate constant for sterol esterification, *r*. Otherwise, they are equal to the respective expressions for the one-pool irreversible model (Eq. 3b) and the two-pool model without considering the ER explicitly (Eq. 28b), when setting *k*_2_ = *k*_− 2_. When comparing Eqs. 40 and 41, one sees that the steady concentration of free sterol in the outer PM leaflet is always larger than that in the inner PM leaflet (i.e., $\overline{S_1}>\overline{S_2}$) for positive rate constants, which is in accordance with net transfer of sterol from the outer to the inner PM leaflet followed by net transfer to the ER (i.e., with *v*_2_,*v*_3_ > 0). The steady state solutions for the sterol in complexes read:

42 $$ \overline{C_1}=\frac{q_4\cdot {v}_1\cdot \left({k}_3\cdot {k}_7+{k}_2\cdot \left({k}_3+{k}_7\right)\right)}{k_2\cdot {k}_3\cdot {k}_7}=\frac{q_4\cdot {v}_1\cdot \left({k}_3\cdot r+{k}_2\right)}{k_2\cdot {k}_3\cdot r}={q}_4\cdot \overline{S_1} $$

43 $$ \overline{C_2}=\frac{q_5\cdot \left({k}_3+{k}_7\right)\cdot {v}_1}{k_3\cdot {k}_7}=\frac{q_5\cdot {v}_1}{k_3\cdot r}={q}_5\cdot \overline{S_2} $$

44 $$ \overline{C_3}=\frac{k_6\cdot {v}_1}{k_7\cdot {k}_{-6}}={q}_6\cdot \overline{S_3} $$

Thus, the relation between complex and free sterol, as found for the 2-pool PM model, is maintained (compare to Eq. 29a, 29b, 29c), and the same relation holds between free and bound sterol in the ER. Thus, not only non-vesicular sterol exchange dynamics but also the kinetics of sterol esterification affects sterol abundance in PM and the ER in this extended model. The latter prediction is in line with experimental observations in which non-vesicular transport of DHE from the PM to the ER, its esterification and storage in lipid droplets was found to require ATP and Are2 activity [27]. The puzzling observation that ATP-depletion inhibited non-vesicular sterol transport from the PM can be understood in light of our findings; sterol esterification requires sterols and activated fatty acids, and the activation of fatty acids with coenzyme A consumes ATP. Despite the fact that we model this complex process by a single irreversible step, any change of Are2 activity or ATP-dependent provision of its substrate should translate into a change of rate constant *k*_7_. The steady state ratio of sterol between the two PM leaflets now reads:

45 $$ {S}_T^i/{S}_T^o=\left(\overline{S_2}+\overline{C_2}\right)/\left(\overline{S_1}+\overline{C_1}\right)=\frac{k_2}{\left({k}_2+{k}_3\cdot r\right)}\cdot \frac{\left(1+{q}_5\right)}{\left(1+{q}_4\right)}={Q}^{\hbox{'}}\cdot \frac{\left(1+{q}_5\right)}{\left(1+{q}_4\right)} $$

Thus, the expression looks very similar to the one for the 2-pool PM model when setting *k*_2_ = *k*_− 2_. (Eqs. 31 and 32). The only difference is the appearance of $r=\frac{k_7}{k_3+{k}_7}$. This weighted rate constant for sterol esterification must be smaller than 1 as long as *k*_3_, *k*_7_ > 0. This situation will prevail in most experimental settings, and Eq. 44 show that the kinetics of sterol esterification in the ER can impact the sterol transbilayer distribution in the PM at steady state. When comparing Eq. 45 with 40 and 41, above, one sees that $\overline{S_1}=\overline{S_2}/Q^{\prime }$, and as Q’ < 1, always, the free sterol pool in the outer leaflet is always larger than that in the inner PM leaflet. An important conclusion from this analysis is that net enrichment of sterol in the inner PM leaflet can only be achieved, if (1 + *q*_5_)/(1 + *q*_4_)> > 1, i.e., when the sterol complexing capacity of the inner leaflet significantly exceeds that of the outer PM leaflet.

Finally, we ask, what is the steady state sterol distribution between PM and ER and how is it affected by the various transport steps. By setting *PM*_T_ and *ER*_T_ the total sterol amount in the PM and ER at steady state, we find:

46 $$ \frac{PM_T}{ER_T}=\frac{\overline{S_1}+\overline{C_1}+\overline{S_2}+\overline{C_2}}{\overline{S_3}+\overline{C_3}}=\frac{k_3\cdot {k}_7\cdot \left(1+{q}_4\right)+{k}_2\cdot \left({k}_3+{k}_7\right)\cdot \left(2+{q}_4+{q}_5\right)}{k_2\cdot {k}_3\cdot \left(1+{q}_6\right)} $$

**Fig. 5:**
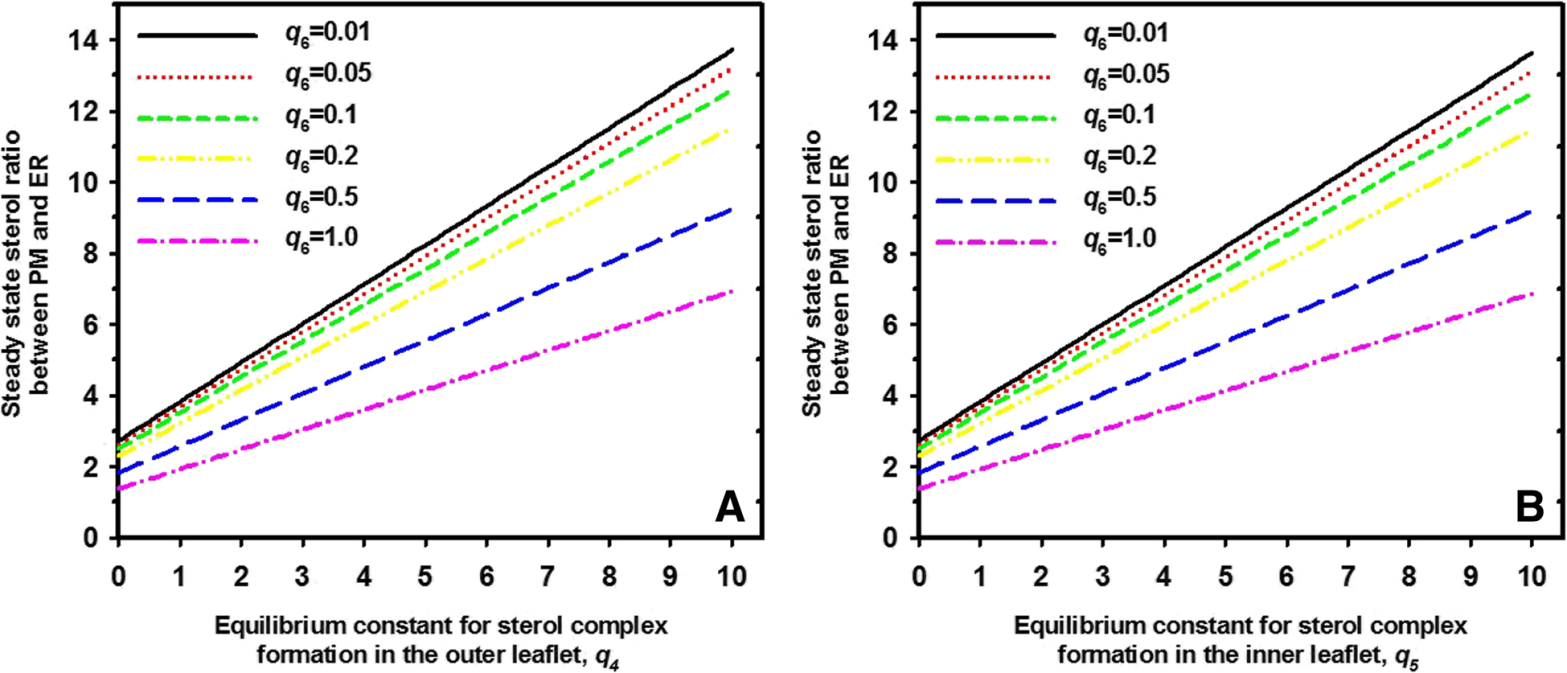
Complex formation of sterol in either PM leaflet compared to the ER sets the steady state sterol ratio between both compartments. Steady state sterol distribution between PM and ER (Eq. 46) as a function of equilibrium constant for sterol complex formation in the outer leaflet (**a** varying *q*_4_) or in the inner leaflet of the PM (**b** varying *q*_5_) for differing values of the equilibrium constant for sterol complex formation in the ER. We set *q*_2_ = *q*_3_ = 1, meaning that forward and backward rate constants for sterol flip flop and for exchange between PM and ER, respectively, are identical. Parameters were *k*_3_ = 0.1 s^− 1^, *k*_2_ = 1 s^− 1^and *K*_7_ = 0.01 s^− 1^, respectively. The equilibrium constant for the complex formation in the corresponding other leaflet was kept constant to *q*_5_ = 0.5 in (**a**) and *q*_4_ = 0.5 in (**b**). See text for further explanations

When plotting this ratio as function of the equilibrium constants for complex formation in the outer or inner PM leaflet, one can determine how complex formation affects sterol partitioning between both organelles at steady state (Fig. 5). As the ER contains mostly unsaturated phospholipids with low propensity to pair with cholesterol, the equilibrium constant for sterol complex formation in the ER, *q*_6_, is set to be low i.e. between *q*_6_ = 0.1 and *q*_6_ = 1. The analysis shows, that preferred sterol complex formation in either the outer or inner PM leaflet result in sterol accumulation in the PM compared to the ER. If the ability of the ER to bind sterol is increased, an even higher complex formation must be ensured in the PM to maintain the steady state sterol distribution between both organelles. For example, for a ratio of 80:20 = 4 between PM and ER, one would need for the equilibrium constant of sterol complex formation in the outer leaflet *q*_4_ = 1.2 and in the inner leaflet *q*_5_ = 0.5 for complex formation in the ER with *q*_6_ = 0.01 (straight black line in Fig. 5a). The same steady state ratio can be obtained if complex formation dominates in the inner PM leaflet (*q*_4_ = 0.5; *q*_5_ = 1.2 for *q*_6_ = 0.01; straight black line in Fig. 5b). However, if the complex formation is stronger in the ER, e.g., *q*_6_ = 1, one would need a much stronger sterol complex formation in either the outer or inner PM leaflet (e.g. *q*_4_ = 4.8; Fig. 5a, pink dashed-dotted line). Thus, the model presented here quantifies the known steady state sterol gradient between PM and ER based on differing sterol membrane interactions in each organelle.

#### Bimolecular sterol complex formation in the PM and ER

The above model made the simplifying assumption, that phospholipids are in excess for the binding such that we obtained pseudo first order rate constants for binding in the outer leaflet, *k*_4_ = *k*_4_*∙P_1_, in the inner leaflet, *k*_5_ = *k*_5_*∙P_2_ and in the ER, *k*_6_ = *k*_6_*∙P_3_ This assumption is only valid under certain assumptions; namely that all phospholipid can form complexes with sterols, and that the mole fraction of sterols in each membrane is low. The latter assumption only holds for relatively large cytoplasmic release combined with low abundance of ergosterol in the medium and/or low transport activity of Aus/Pdr11, both resulting in low import fluxes, *v*_1_. The foregoing assumption only applies to cases, where all phospholipids in a membrane would interact equally with sterols, which is likely not realistic for a cellular membrane. Instead, membrane lipids bearing saturated acyl chains would interact preferentially (i.e., would form condensed complexes with ergosterol or cholesterol), while unsaturated phospholipids might avoid direct contact to sterols [53, 55]. A more realistic scenario is to consider complex formation explicitly, which requires solving the steady state system for a bimolecular complexation rate in each membrane pool. This gives the following transport rates: *v*_1_ = const., *v*_2_ = *k*_2_ ⋅ (*S*_1_ − *S*_2_), *v*_3_ = *k*_3_ ⋅ (*S*_2_ − *S*_3_), *v*_4_ = *k*_4_ ⋅ *S*_1_ ⋅ *P*_1_ − *k*_−4_ ⋅ *C*_1_, *v*_5_ = *k*_5_ ⋅ *S*_2_ ⋅ *P*_2_ − *k*_−5_ ⋅ *C*_2_, *v*_6_ = *k*_6_ ⋅ *S*_3_ ⋅ *P*_3_ − *k*_−6_ ⋅ *C*_3_ and *v*_7_ = *k*_7_ ⋅ *S*_3_ Here, *P*_1_, *P*_2_ and *P*_3_ are the sterol binding partners for complex formation in each pool, assumed to be saturated phospholipids for simplicity. A stoichiometry of 1:1, sterol:phospholipid is chosen to simplify the analysis and as justified by studies on model membranes of certain lipid compositions [55, 63]. This bi-molecular model can account for high sterol mole fraction and also for limiting availability of phospholipid binding partners, a key ingredient of the condensed complex model. In fact, condensed complexes have been invoked to explain the phase behavior of binary sterol-phospholipid complexes but also of ternary mixtures of sterol, unsaturated and saturated phospholipids, in which only the latter are assumed to interact preferentially (be ‘reactive’ according to [53, 55]). With this extension the resulting ODE for our steady state system reads:

47a-i $$ {\displaystyle \begin{array}{l}\frac{dS_1}{dt}={v}_1-{v}_2-{v}_4={k}_1\cdot {S}_0-\left({k}_2+{k}_4\cdot {P}_1\right)\cdot {S}_1+{k}_2\cdot {S}_2+{k}_{-4}\cdot {C}_1\\ {}\frac{dS_2}{dt}={v}_2-{v}_3-{v}_5={k}_2\cdot {S}_1-\left({k}_2+{k}_3+{k}_5\cdot {P}_2\right)\cdot {S}_2+{k}_{-5}\cdot {C}_2+{k}_3\cdot {S}_3\\ {}\frac{dS_3}{dt}={v}_3-{v}_6-{v}_7={k}_3\cdot {S}_2-\left({k}_3+{k}_6\cdot {P}_3+{k}_7\right)\cdot {S}_3+{k}_{-6}\cdot {C}_3\\ {}\frac{dC_1}{dt}={v}_4={k}_4\cdot {S}_1\cdot {P}_1-{k}_{-4}\cdot {C}_1\\ {}\frac{dC_2}{dt}={v}_5={k}_5\cdot {S}_2\cdot {P}_2-{k}_{-5}\cdot {C}_2\\ {}\frac{dC_3}{dt}={v}_6={k}_6\cdot {S}_3\cdot {P}_3-{k}_{-6}\cdot {C}_3\\ {}\frac{dP_1}{dt}=-{v}_4=-{k}_4\cdot {S}_1\cdot {P}_1+{k}_{-4}\cdot {C}_1\\ {}\frac{dP_2}{dt}=-{v}_5=-{k}_5\cdot {S}_2\cdot {P}_2+{k}_{-5}\cdot {C}_2\\ {}\frac{dP_3}{dt}=-{v}_6=-{k}_6\cdot {S}_3\cdot {P}_3+{k}_{-6}\cdot {C}_3\end{array}} $$

At first glance, this system consisting of nine coupled ODEs looks rather complicated and apparently tedious to solve, but it simplifies considerably for the steady state scenario; since concentrations do not change at steady state, all expressions are set to zero. This implies *v*_4_ = *v*_5_ = *v*_6_ = 0, such that the steady state concentration of active sterol in the PM and ER can be calculated from the much simpler system, where we get for the outer and inner leaflet of the PM again:

48 $$ \overline{S_1}={v}_1\cdot \left(\frac{1}{k_2}+\frac{1}{k_3}+\frac{1}{k_7}\right) $$

49 $$ \overline{S_2}=\frac{v_1\cdot \left({k}_3+{k}_7\right)}{k_3\cdot {k}_7} $$

This is identical to the expressions derived for the linearized model (Eqs. 38 and 39, above), showing that bimolecular complex formation of sterol in each leaflet does not affect the steady state concentrations of active sterol. The steady state amount of active sterol in the ER is also identical to the linear model and reads:

50 $$ \overline{S_3}=\frac{v_1}{k_7} $$

One also sees that the system has only one non-vanishing steady state flux, which is again *v*_1_ = *v*_2_ = *v*_3_ = *v*_7_. This is an important and somehow surprising result; flux of active sterol through the Aus1/Pdr11 importers, flip-flop across the PM, transport to the ER and esterification become equal at steady state, while the complexed sterol pools in each membrane do not explicitly contribute to this flux. So, how are sterol complexes related to active sterol at steady state?

#### Complexes of sterol and phospholipid give rise to non-linear sterol pool sizes in the PM and ER

To derive this we make use of a conservation relation, which is that the concentration of phospholipid in the unbound form and in complexes is preserved in each pool. That means, the total pool of sterol binding partners in the outer leaflet (*P*_T1_), in the inner leaflet (*P*_T2_) and in the ER (*P*_T3_) is constant, such that.

51 $$ \overline{C_i}+\overline{P_i}={P}_{Ti},\kern1.00em \forall \kern0.5em i=1,2,3 $$

With that used in Eq. 47a-i e, f, one can derive the steady state concentrations of sterol in complexes as

52 $$ {\displaystyle \begin{array}{l}\overline{C_1}=\frac{q_4\cdot \overline{S_1}\cdot {P}_{T1}}{k_4\cdot \overline{S_1}+{k}_{-4}}=\frac{v_1\cdot {k}_4\cdot {P}_{T1}\cdot \left({k}_3\cdot {k}_7+{k}_2\cdot \left({k}_3+{k}_7\right)\right)}{v_1\cdot {k}_3\cdot {k}_4\cdot {k}_7+{k}_2\cdot \left({k}_3\cdot {k}_7\cdot {k}_{-4}+{v}_1\cdot {k}_4\cdot \left({k}_3+{k}_7\right)\right)}\\ {}\kern1.50em \begin{array}{cc}& \end{array}=\frac{v_1\cdot {q}_4\cdot {P}_{T1}\cdot \left({k}_3\cdot r+{k}_2\right)}{v_1\cdot {q}_4\cdot \left({k}_3\cdot r+1\right)+{k}_2\cdot {k}_3\cdot r}\end{array}} $$

53 $$ \overline{C_2}=\frac{q_5\cdot \overline{S_2}\cdot {P}_{T2}}{k_5\cdot \overline{S_2}+{k}_{-5}}=\frac{v_1\cdot {k}_5\cdot {P}_{T2}\cdot \left({k}_3+{k}_7\right)}{k_3\cdot {k}_{-5}\cdot {k}_7+{v}_1\cdot {k}_5\cdot \left({k}_3+{k}_7\right)}=\frac{v_1\cdot {q}_5\cdot {P}_{T2}}{k_3\cdot r+{v}_1\cdot {q}_5} $$

54 $$ \overline{C_3}=\frac{q_6\cdot \overline{S_3}\cdot {P}_{T3}}{k_6\cdot \overline{S_3}+{k}_{-6}}=\frac{v_1\cdot {k}_6\cdot {P}_{T3}}{k_{-6}\cdot {k}_7+{v}_1\cdot {k}_6}=\frac{v_1\cdot {q}_6\cdot {P}_{T3}}{k_7+{v}_1\cdot {q}_6} $$

**Fig. 6:**
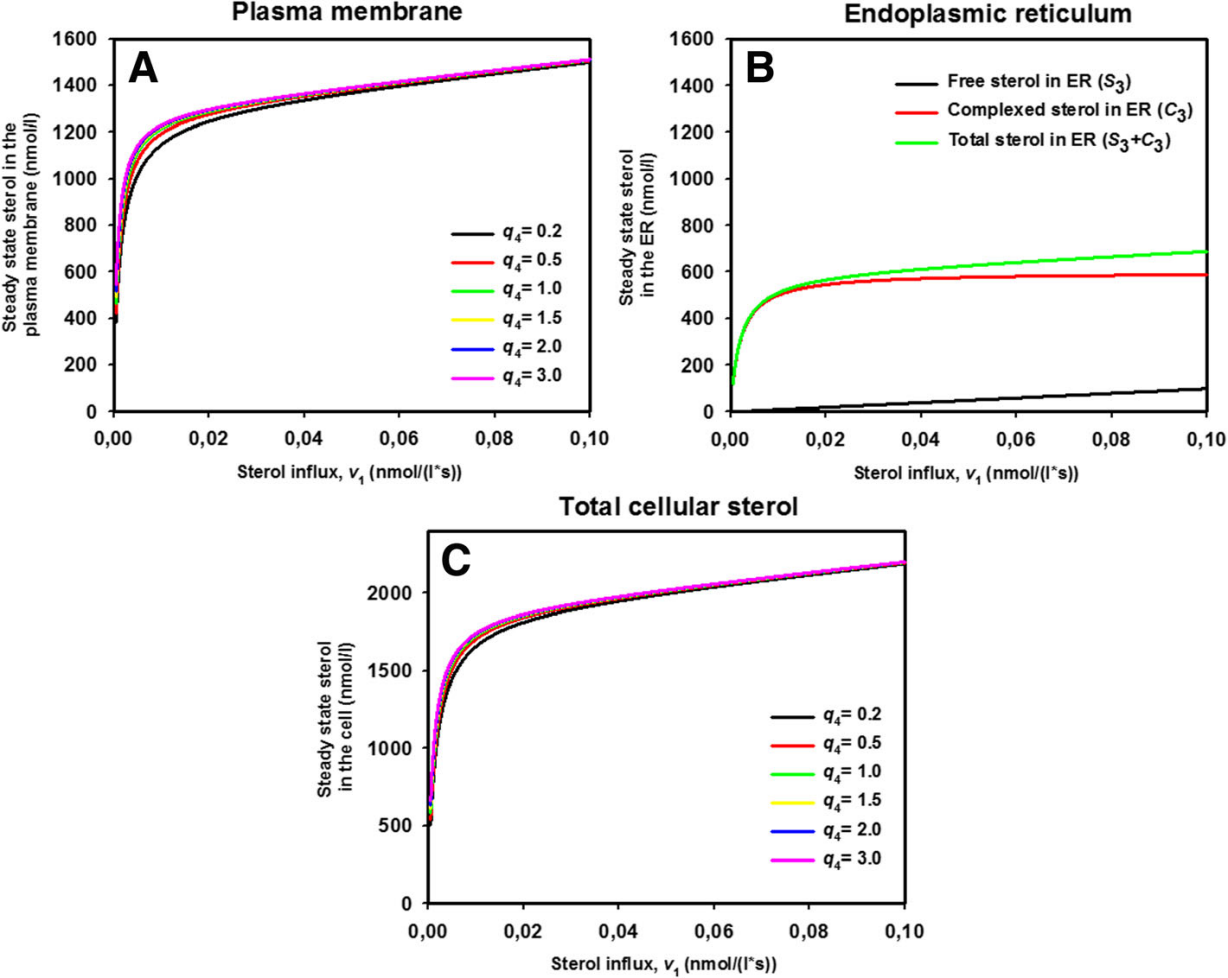
Bi-molecular complex formation of sterol with phospholipids gives rise to combined hyperbolic and linear sterol influx into the PM and the ER. Accounting explicitly for sterol complex formation with a limited number of phospholipids in the outer PM leaflet (*P*_T1_ = 300 nM), the inner PM leaflet (*P*_T2_ = 1000 nM) or the ER (*P*_T1_ = 600 nM) results in a bi-phasic dependence of sterol abundance in each membrane pool on sterol influx (*v*_1_, varied from 0.5 pM∙s^− 1^ to 0.33 nM∙s^− 1^). The highest flux value is 10times that calculated for yeast cells using realistic values of catalytic activity and abundance of the sterol importers Aus1/Pdr11 (see text). Thus, it would represent a 10fold increased expression level of Aus1/Pdr11. Sterol abundance was calculated using Eqs. 48–50 and 52–54 for different values of the equilibrium constant for sterol complexation in the outer PM leaflet, *q*_4_, and plotted as function of sterol in flux for the PM (**a**) and for total cellular sterol (**c**). For the ER pool (which is independent of *q*_4_, see Eqs. 50 and 54), the free (‘active’; black line in **b**), complexed (red line in **b**) and total sterol (green line in **b**) was plotted as function of sterol influx. Other parameters were *k*_2_ = 0.1 s^− 1^, *k*_3_ = 0.01 s^− 1^, *q*_5_ = 1.0 and *q*_6_ = 0.5

Similar expressions hold for the steady state concentration of non-complexed phospholipid in each pool (not shown). One sees from Eqs. 48 to 50 and the right-hand side of Eqs. 52 to 54 that sterol abundance in each membrane pool is independent of the kinetic rate constants for sterol complex formation and only depends on their ratio, i.e., the respective equilibrium constants *q*_4_, *q*_5_ and *q*_6_. Thus, the exact kinetics of sterol complex formation does not impact the sterol pool sizes at steady state. To use this result for simulating the DHE uptake experiments (see above and [31]), we consider the high substrate regime and set *v*_1_^MM^ = *v*_max_ in a range covering a large range of transporter expression and activity including the calculated value of *v*_1_^MM^ = 0.033 nmol/(l∙s), i.e. we set *v*_1_ = 0.0005–0.1 nmol/(l∙s). Sterol binding partners are set to *P*_T1_ = 300 nmol/l in the outer PM leaflet, *P*_T2_ = 1000 nmol/l in the cytoplasmic half of the PM and *P*_T3_ = 600 nmol/l. This is only a fraction of total phospholipid in each pool, which, in case of the PM, surmounts for example to around 1300 nmol/l in each PM leaflet [20, 31]. Thus, we assume that the remaining membrane lipids do not contribute to complex formation and are considered as ‘unreactive’ phospholipids, following the nomenclature of [55]. The equilibrium constant for complex formation in the outer PM leaflet is varied between *q*_4_ = 0.2 to 3.0, while that for the inner leaflet is set to *q*_5_ = 1.0 (Fig. 6). These values compare favorably to those used in simulation of condensed complexes in model lipid systems (i.e. two-phase coexistence and rapid release of sterol from lipid monolayers have been observed for equilibrium constants of sterol complexes in the range of K = 0.5–2.0 [15, 52, 53]. However, it has to be noted that the latter parameters were defined based on experiments in lipid monolayers, in which the equilibrium constant for complex formation is a function of surface pressure, something not considered in our model [53].

One sees, that the abundance of imported sterol in the PM (Fig. 6a), the ER (Fig. 6b) and the whole cells (Fig. 6a) depend for low fluxes in a hyperbolic fashion and for higher fluxes in a linear fashion on the sterol influx, *v*_1_. For higher propensity to form complexes (here varied for the outer PM leaflet, *q*_4_), the initial rise in uptake (i.e. for low flux values) is larger than for small *q*_4_. For larger flux values, i.e., above ca. *v*_1_ = 0.5 nmol/(l∙s), these differences vanish, as exemplified for PM and total sterol (Fig. 6a and c). To analyze this further the abundance of free and complexed sterol as function of sterol influx was plotted separately, here for the ER pool (Fig. 6b). While sterol complexes depend hyperbolically on *v*_1_, the active sterol pool depends linearly on sterol influx (compare red, $\overline{C_3}$, and black curve, $\overline{S_3}$, in Fig. 6b and see Eq. 54 and 50, above). Thus, being the sum of the respective active and complexed sterol pools, total sterol in each compartment becomes a combination of a hyperbolic and a linear function of sterol influx, *v*_1_. A straightforward experimental test of this prediction would be to determine steady state levels of the fluorescent ergosterol analog DHE in the PM and the ER of *Δhem1* yeast cells for varying expression levels of Aus1/Pdr11. In fact, expression of these transporters is under control of the transcription factor Upc2, which in turn senses intracellular sterol abundance [64]. Upc2 was shown to bind ergosterol and DHE and to move to the nucleus to initiate transcription upon sterol depletion [65]. Thus, transcriptional regulation of sterol influx, *v*_1_, is likely an important control strategy used by yeast cells under anaerobic growth conditions.

#### Complexes of sterol and phospholipid can control sterol distribution in the PM and between PM and ER

**Fig. 7:**
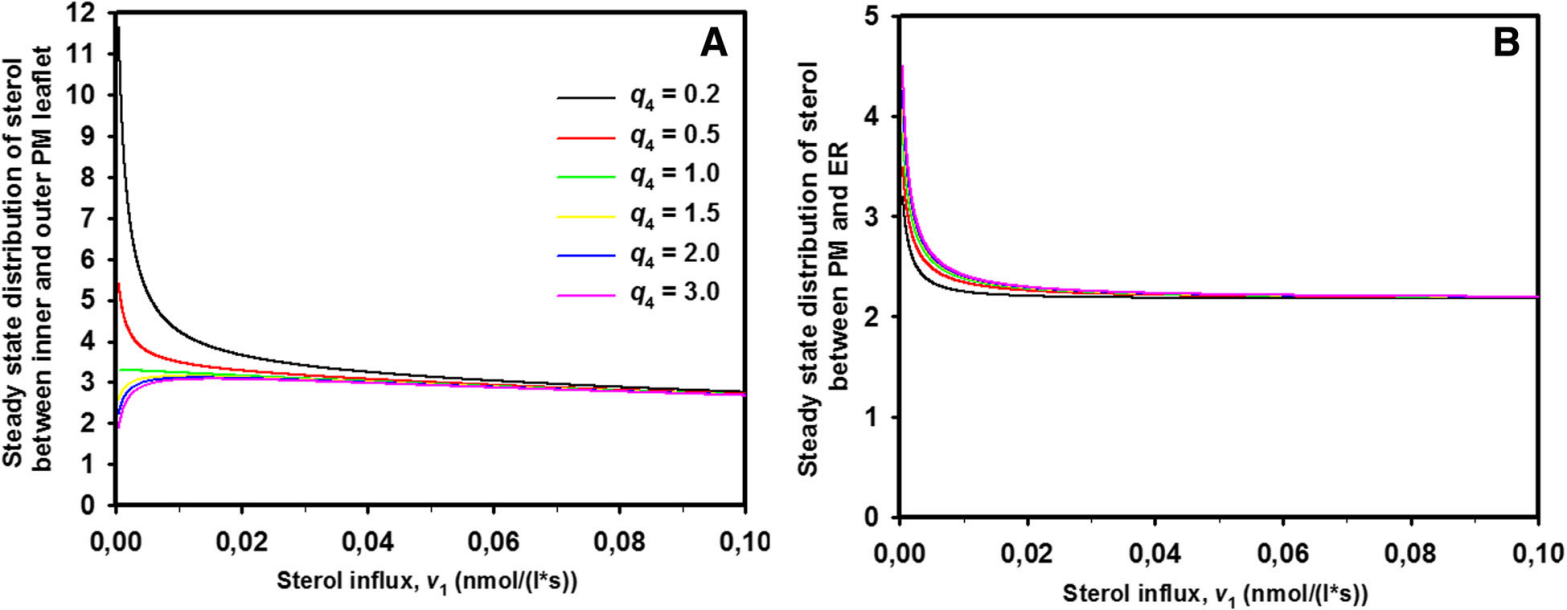
Low sterol influx exerts control over sterol distribution between membrane pools for bi-molecular complex formation. Accounting explicitly for sterol complex formation with a limited number of phospholipids in the outer PM leaflet (*P*_T1_ = 300 nM), the inner PM leaflet (*P*_T2_ = 1000 nM) or the ER (*P*_T1_ = 600 nM) results in highly non-linear dependence of sterol distribution between the two PM leaflets (**a**) and between PM and ER (**b**) on sterol influx, particularly for low influx values. Here, the equilibrium constant for sterol complexation in the outer PM leaflet, *q*_4_, was varied as indicated in panel (**a**). All other parameters were set as described in legend to Fig. 6, above

In previous sections, we showed that the steady state transbilayer distribution of sterol between the two PM leaflets is independent of sterol influx, in yeast mediated by Aus1/Pdr11. Does this also hold true for the non-linear model including sterol complex formation? Sterol distribution between the two PM leaflets can be calculated from Eqs. 48–50 and 52–54, respectively, showing that it depends non-linearly on *v*_1_ for small influx values (Fig. 7a). Our model predicts that the extent of sterol import into cells directly affects the transbilayer sterol distribution as long as sterol influx is low compared to the ability of complex formation in each leaflet. For larger flux values (i.e., *v*_1_ ≥ 0.5 nmol/(l∙s)), sterol distribution between the PM leaflets becomes largely independent of sterol influx and of the sterol complex forming capability of each leaflet (in Fig. 7a shown for varying *q*_4_). Instead the plateau sterol ratio in the PM is primarily set by the abundance of binding partners, e.g. saturated phospholipids in the outer and inner PM leaflet. Thus, our model predicts that preferred location of sterol binding partners in the inner PM leaflet is instrumental for steady state sterol enrichment in this leaflet. To recapitulate the experimentally observed sterol asymmetry of about 80:20 in favor of the inner leaflet, we set phospholipid binding partners in the outer and inner leaflet to *P*_1_ = 300 nmol/l and *P*_2_ = 1 μmol/l, respectively (Fig. 7a). This corresponds to about 20 and 67% of all phospholipids in the outer and inner PM leaflet, respectively. Even though condensed complexes have been shown to form between sterol and outer leaflet lipids, like sphingolipids as well as inner leaflet lipids, like PS [15, 53], it is currently not supported by experiments, that the inner PM leaflet contains significantly more sterol-interacting lipid species compared to the outer leaflet. One can assume that additional factors, like sterol-binding proteins cause net enrichment of sterol in the cytoplasmic leaflet. Further experimental studies are required to identify and characterize such factors in living cells.

Our model can also recapitulate the experimentally observed 70:30 distribution of ergosterol or DHE between PM and ER for reasonable sterol influx (Fig. 7b). In addition, the model predicts low steady state sterol influx (*v*_1_ ≤ 0.01 nmol∙l^− 1^∙s^− 1^) causes a strong dependency of the ability to form sterol complexes in the ER relative to that in the PM on the steady state distribution of sterol between both compartments. Here, the equilibrium constant for sterol complex formation in the outer PM leaflet, *q*_4_, was varied relative to that in the ER, *q*_6_, Fig. 7b, but the same qualitative result is obtained by varying *q*_5_, the equilibrium constants for sterol complexes in the inner PM leaflet (not shown). For larger sterol influx, (*v*_1_ ≥ 0.05 nmol∙l^− 1^∙s^− 1^), the steady state sterol ratio between PM and ER becomes completely independent of any sterol complex formation propensity (Fig. 7b). This is, because the active but not the complexed sterol pool moves freely and contributes to the steady state flux between both organelles. As a direct consequence of this result one can predict, that the expression levels of Aus1/Pdr11 in the PM and of Are2 in the ER exert coordinated control over the steady state ratio of sterol between both organelles as long as their activity does not exceed the sterol complexing capability of the PM. This outcome of our model is in line with some recent results regarding the role played by Aus1/Pdr11 in yeast sterol transport, in which a direct effect of Aus1/Pdr11 activity not only on uptake but also on sterol esterification in the ER has been reported [21, 66]. In one of these studies, even a physical interaction between Aus1/Pdr11 and Are2 at close PM-ER membrane appositions has been suggested [66]. Such an interaction could facilitate the coordination of sterol uptake and esterification via allosteric mechanisms, thereby fine tuning metabolic control of sterol homeostasis in yeast.

#### Sterol-phospholipid complexes cause a threshold-like dependence of the ER sterol pool size on PM sterol

**Fig. 8:**
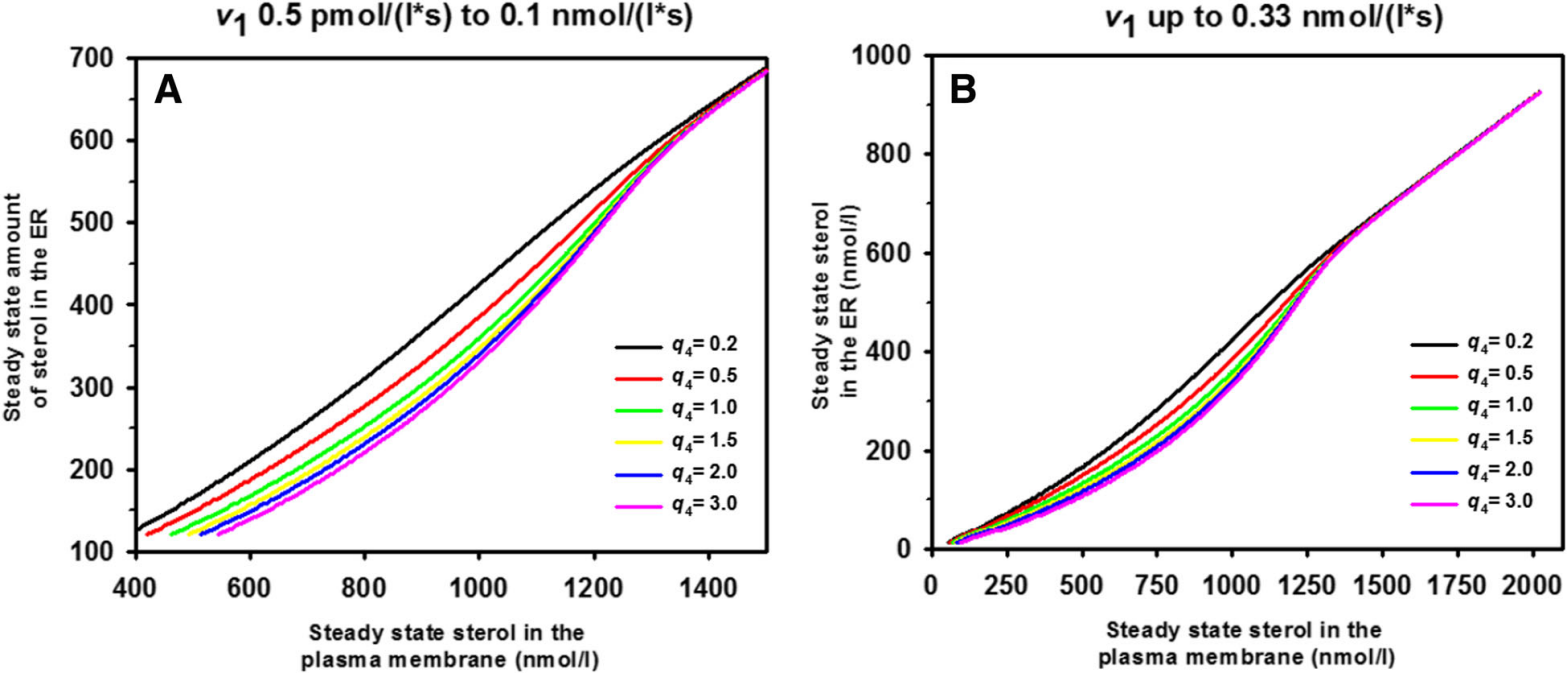
Bi-molecular complex formation of sterol with phospholipids gives rise to non-linear dependence of ER sterol on sterol abundance in the PM. Sterol abundance in the ER was plotted as function of sterol abundance in the PM for low influx values (**a**) and for higher influx values (**b**). The equilibrium constant for sterol complexation in the outer PM leaflet, *q*_4_, was varied as indicated. All parameters were set as described in legend to Figs. 6 and 7, above. See text for further explanations

Increased cholesterol in the PM of mammalian cells triggers several physiological responses in the ER above certain threshold values (reviewed in [8, 14]). For example, cholesterol’s concentration in the ER depends non-linearly on that in the PM, and all of cholesterol added to cells beyond complexing capacity of the PM moves to the cytoplasmic membranes in fibroblasts [18, 41, 67, 68]. Activation of ACAT which esterifies cholesterol for storage in LDs in macrophages, takes also place upon expanding cholesterol levels beyond some threshold value, and that has been shown to be accompanied by rapid sterol influx from the PM [17, 43, 69]. Our bi-molecular model reconciles such observations (Fig. 8): for certain parameter combinations we found a steep rise in ER sterol once sterol levels in the PM passed some threshold value due to increasing sterol influx, *v*_1_ (i.e., for strong preference of complex formation; q_4_, q_5_ ≥ 10, not shown). Thus, our model can describe threshold-like responses, but unfortunately, for such parameters, the steady state sterol ratio between PM and ER could not reconcile the experimentally determined 70:30 distribution in yeast. Thus, extreme threshold effects are either not present in the yeast system or require extensions of our model, i.e., probably cooperative phospholipid-sterol interactions would need to be included to account for that in a more physiological range of parameters. However, since the algebra gets more involved and strong thresholds have not been observed for yeast so far, this has not been considered further. Furthermore, the steepness of the experimentally observed threshold effect depends not only on the sterol complexing capability of the involved lipid species but also to some extent on the method used for threshold detection. 12That is, protein sensors, like perifringolysin or anthrolysin show highly cooperative membrane binding once sterol mole fractions in the bilayer exceed some threshold values [18, 59, 70]. Thus, the extent of non-linearity of a given response might depend on the experimental system and the method of its interrogation.

For parameters reconciling all experimental observations in the yeast system, the non-linear rise in ER sterol upon increase in PM sterol is predicted to be milder (Fig. 8a). Interestingly, this non-linear dependence of ER sterol abundance on sterol in the PM is only seen for low sterol influx values, where the hyperbolic sterol complex formation dominates. Plotting ER sterol as function of PM sterol over a larger range of sterol influx values reveals that this threshold actually describes a transition (Fig. 8b): for low sterol influx, complex formation can keep up with incoming new sterol. Once all binding phospholipids are consumed, i.e., beyond the stoichiometric capacity of complex formation, ER sterol depends linearly on PM sterol (Fig. 8b, beyond ca. 1.4 μmol/l PM sterol). This is, because from this point on each newly imported sterol molecule only feeds into the active sterol as the complexation capacity of the PM has been exceeded. Accordingly, we see a linear relationship between ER and PM sterol for larger sterol influx, *v*_1_. The latter has – to our knowledge – not been reported, yet; neither in mammalian cells nor in yeast. This can have two reasons; first, steady state experiments are rarely carried out, even though this is in principal straightforward in yeast bearing a Δ*hem1* mutation, as outlined above. Instead, many experiments are carried out after membrane fractionation, e.g., reports using cholesterol-binding cytolysin derivatives or those involving radioactive sterols [9, 18, 70]. Such experiments can result in thermodynamic equilibration of pool sizes such that they cannot be compared directly to the steady state analysis presented here. Second, cells might avoid exceeding the threshold for active sterol too far and initiate homeostatic mechanisms to reset active sterol pool sizes below the threshold value [18, 59].

## Discussion

The majority of cholesterol or ergosterol resides in the PM with low sterol abundance in the ER at steady state [14, 18, 20, 31, 41]. Recent studies in mammalian cells and yeast have shown that the majority of sterols in the PM reside in its inner leaflet [31, 71, 72]. However, some earlier studies report sterol enrichment in the outer compared to the inner leaflet, as recently reviewed in [73]. Technical challenges in measuring sterol distribution in the PM exactly have been suggested to be responsible for such discrepancies [31, 71, 73]. Our steady state sterol transport model predicts that other factors such as varying sterol import flux, cytoplasmic sterol release and rate of sterol esterification in the ER can impact the abundance and transbilayer distribution of ergosterol in the yeast PM. Sterols in the PM can rapidly equilibrate with the ER and other organelles via non-vesicular transport, both in mammalian cells [74–76] and in yeast [9, 20, 27, 29]. The magnitude of non-vesicular sterol transfer from the PM can act as homeostatic mechanism for sterol abundance not only in the ER but also in recycling endosomes and lysosomes, which both have been shown to equilibrate their sterol pools rapidly with that in the PM [77–80]. Analogs of cholesterol and ergosterol show also fast transbilayer migration (flip-flop) in most model membrane studies [32, 33, 81, 82]. It is ad hoc not clear, how such strong sterol gradients between membranes or between their two leaflets can be maintained in the presence of high sterol fluxes. Here, we have used a mathematical approach and developed a simple steady state model to understand the key features of sterol flux across the yeast PM, thereby explaining apparently contradicting observations.

## Conclusions and future perspectives

Key predictions of our model analysis are:

1. Influx of sterol into yeast cells under anaerobic growth conditions via ABC transporters sets the rates for sterol flip-flop across the PM, non-vesicular sterol transport to the ER and sterol esterification at steady state (i.e., there is only one non-equilibrium flux, *v*_1_ = *J*, at steady state).
2. The attainable transbilayer sterol asymmetry at steady state is always (slightly) smaller than the equilibrium ratio (Fig. 2). Sterol esterification in the ER controls cytoplasmic sterol release and thereby impacts sterol abundance and transbilayer distribution in the PM.
3. The steady state flux is an exponentially decaying function of the non-equilibrium chemical potential difference of sterol between both PM leaflets (Fig. 3). Thus, apart from ATP-dependent sterol import, ATP-consuming sterol esterification by Are2 in the ER keeps the system out of equilibrium, thereby exerting metabolic control over cellular sterol homeostasis.
4. Rapid sterol exchange between both PM leaflets (Fig. 4) and between PM and ER (Fig. 5) can be accounted for by a 2-pool model, in which sterol exists free for exchange (‘active’) or in complexes with phospholipids or proteins in both membranes.
5. For equal sterol flip-flop rates, enrichment of ergosterol in one PM leaflet requires its preferred complex formation in that leaflet (Fig. 5). In fact, the concentration of free, non-complexed sterol is larger in the outer than in the inner PM leaflet to ensure net transfer in the import direction. As a consequence, experimental methods which primarily detect free, non-complexed sterol could misinterpret the transbilayer distribution of sterols in the PM. The abundance of sterol binding partners, such as saturated phospholipids, determines the attainable sterol asymmetry in the PM.
6. Sterol abundance in the cells and their membranes increases hyperbolically for low influx rates due to formation of sterol-phospholipid complexes (Fig. 6). For higher import rates, the complex forming capability of the PM is exceeded, and a linear relationship between sterol abundance and influx becomes apparent.
7. The steady state ratio of sterol between both PM leaflets as well as between the PM and ER is predicted to dependent in a non-linear manner on activity or abundance of Aus1/Pdr11 in the PM and Are2 in the ER (Fig. 7). Once the sterol influx exceeds the capacity of sterol complex formation in the PM, changes of import efficiency do not affect sterol distributions significantly.
8. Complex formation leads to a non-linear increase in sterol content of the ER as function of sterol in the PM (Fig. 8). For high sterol influx, a linear relationship between PM and ER sterol is re-established, as only active (free) sterol contributes to steady state flux between both compartments.

These are testable predictions for future experiments, in which the steady state sterol distribution between both PM leaflets as well as between PM and ER is assessed, e.g. using fluorescence imaging of the close ergosterol analog DHE. By varying the abundance of Aus1/Pdr11, whose expression is under control of the transcription factor Upc2 [65], one will be able to determine the regulatory mechanisms underlying sterol import under anaerobic growth conditions in yeast. Transport-coupled esterification of DHE in the ER and its storage in LDs can be studied by a combination of biochemical and imaging experiments in cells with varying expression level of Are2 [27]. This should translate into varying rate constant *k*_7_ of our model. By varying the abundance and/or distribution of sphingolipids and PS using chemical inhibitors, mutants of enzymes involved in lipid synthesis or knock-outs of PM phospholipid flippases, one will be able to determine, to what extent lipids contribute to sterol complex formation in the PM and thereby to maintenance of the sterol gradient between PM and ER. Furthermore, a variety of sterol or sphingolipid sensing cytolysins could be expressed as fluorescence-tagged constructs and studied in concert with DHE by multi-color fluorescence microscopy [83]. Assuming passage of the low-molecular weight proteins through the yeast cell wall, their binding to the outer PM leaflet could be even studied after appropriate fluorescence labeling. This could even allow for dissection of sphingolipid-sterol interactions in the outer PM leaflet of yeast cells using ostreolysin A, a cytolysin recently described as molecular sensor for sphingomyelin-cholesterol complexes [16]. Combining such experiments with chemical inhibition of biosynthesis of some lipid species will provide valuable insight into sterol interactions in native membranes. This could allow for a direct test of another hypothesis, namely that ergosterol is supposed to segregate from tightly packed sphingolipids in the yeast PM [57].

An extension of the model in future studies could include accounting for cooperative interactions between sterol and phospholipid in complexes, which could even better describe threshold-like transitions in sterol release from the PM, as observed for mammalian cells [17, 19, 41, 43, 44]. Cooperative sterol complexation has been suggested based on cholesterol-phospholipid phase diagrams in model lipid systems, and such a process could be included in our model by introducing a new parameter for oligomerization [15, 52, 55]. In addition, the stoichiometry of complex formation between sterol and phospholipid might be varied using m, *n* > 1 in the rates *v*_4_, *v*_5_ and *v*_6_ (Eq. 35), as other stoichiometries than 1:1 have been suggested in several studies [15, 54, 63, 84]. Such an extension will complicate the algebra significantly calling for numerical solutions, but it might be an approach to describe highly non-linear flux responses for realistic parameter combinations in both, yeast and mammalian cells.

Finally, it should not go unnoticed, that our model is a simplification of the real biochemical scenario, and that many aspects of the complex problem of intracellular cholesterol transport are not considered. Non-steady state sterol biosynthesis and steryl ester hydrolysis will certainly contribute to sterol homeostasis but have not been considered in the model presented here. Allosteric activation of sterol esterification by sterol abundance in the ER is likely to play a role as well, but has not been included in our model, either. Yeast and mammalian cells have to operate under a variety of environmental conditions, making that their homeostatic control mechanisms have to deal with several inputs, for example, by adjusting the expression of proteins in a given metabolic pathway. Anaerobic sterol uptake in yeast is under tight control of the expression factor Upc2, which responds to a decrease in cytosolic ergosterol by translocation to the nucleus for initiating expression of Aus1/Pdr11 and other proteins [65]. Such feedback mechanisms have not been considered here, but could be included in future studies. Similarly, transport and metabolism of sterols and other lipids can change throughout the yeast cell cycle, for example prior to and after diauxic shift or during growth resumption and in the stationary state [85, 86]. Also, steryl esters in LDs can be utilized by other pathways, such as by ingestion into the vacuole (the yeast lysosome) during starvation in a process called lipophagy [87, 88]. Metabolic adjustments such as utilization of LDs are carried out in concert with the PM and the vacuole via target of rapamycin 1 and 2 (TORC1 and TORC2), two kinases regulating metabolic programs under various environmental cues [86, 89]. An important but challenging strategy could be to include these aspects into our model in the future. Finally, many of the rate constants used here have been estimated based on literature values, which cannot replace a proper parametrization of the model. For that, one would need to carry out kinetic experiments and fit time-dependent solutions of the model to the data. This will be attempted in future studies.

**Table:** 

**Parameter symbol**	**Parameter function**	**Explored parameter values**	**Lipid pool symbol**	**Lipid pool definition**
*v*_1_ = *J*	**Sterol influx at steady state**	0.5 pmol·l^− 1^·s^− 1^ to 0.1 nmol·l^− 1^·s^− 1^	${S}_1^{eq}$	**Sterol in outer PM leaflet at equilibrium**
*v*_max_	**Maximal activity of Aus1/Pdr11**	0.5 pmol·l^− 1^·s^− 1^ to 0.33 nmol·l^− 1^·s^− 1^	${S}_2^{eq}$	**Sterol in nner PM leaflet at equilibrium**
E_T_	**Concentration of Aus1/Pdr11**	0.2 nmol·l^− 1^·	$\overline{S_1}$	**Freely mobile sterol pool in outer PM leaflet at steady state**
${k}_M=\frac{m_{-1}+{m}_2}{m_1}$	**Michaelis-Menten constant of sterol import**	NA	$\overline{S_2}$	**Freely mobile sterol pool in inner PM leaflet at steady state**
*k*_1_	**Import rate constant**	NA	$\overline{S_3}$	**Freely mobile sterol pool in ER at steady state**
*k*_−1_	**Export rate constant**	NA	$\overline{C_1}$	**Sterol-phospholipid complexes in outer PM leaflet at steady state**
*k*_2_	**Flip rate constant**	0.1–10 s^− 1^	$\overline{C_2}$	**Sterol-phospholipid complexes in inner PM leaflet at steady state**
*k*_−2_	**Flop rate constant**	0.1–1 s^− 1^	$\overline{C_3}$	**Sterol-phospholipid complexes in ER at steady state**
*k*_3_	**Rate constant for non-vesicular transport**	0.01–1 s^− 1^	*P*_T1_	**Phospholipid available for complexes in outer PM leaflet**
*k*_4_	**Rate constant for sterol-phl. complexation in outer PM leaflet**	0.001–1 s^− 1^	*P*_T2_	**Phospholipid available for complexes in inner PM leaflet**
*k*_−4_	**Rate constant for sterol-phl.** **dissociation in outer PM leaflet**	0.001–1 s^− 1^	*P*_T3_	**Phospholipid available for complexes in ER**
*k*_5_	**Rate constant for sterol-phl.** **complexation in inner PM leaflet**	0.001–1 s^− 1^	*S*_T_ = *PM*_T_	**Total sterol in PM**
*k*_−5_	**Rate constant for sterol-phl.** **dissociation in inner PM leaflet**	0.001–1 s^− 1^	*ER*_T_	**Total sterol in ER**
*k*_6_	**Rate constant for sterol-phl. complexation in ER**	0.001–1 s^− 1^	*Q*	**Ratio of free sterol between PM leaflets in Eq.** **1**
*k*_−6_	**Rate constant for sterol-phl. dissociation in ER**	0.001–1 s^− 1^	*Q’*	**Ratio of free sterol between PM leaflets in Eq.** **44**, **5**
*k*_7_	**Rate constant for sterol esterification in ER**	0.001–1 s^− 1^	${S}_T^o$	**Total sterol in outer PM leaflet**
*q*_2_	**Equilibrium constant for sterol flip-flop**	0.01–10	${S}_T^i$	**Total sterol in inner PM leaflet**
*q*_3_	**Equilibrium constant for sterol transport between PM and ER**	0.01–10	*S*_0_	**Total sterol in medium**
*q*_4_	**Equilibrium constant for complexes in outer PM leaflet**	0.01–10		
*q*_5_	**Equilibrium constant for complexes in inner PM leaflet**	0.01–10		
*q*_6_	**Equilibrium constant for complexes in ER**	0.01–1.0		
*r*	**Fractional rate constant for sterol esterification in ER**	NA		
${C}_{k_i}^J$	**Flux control coefficient with respect to parameter** ***k***_***i***_	1		
${C}_{k_i}^{S_j}$	**Concentration control coefficient with respect to parameter** ***k***_***i***_	1		

## Data Availability

The datasets used and/or analyzed during the current study are available from author on request.

## Abbreviations

DHE: Dehydroergosterol
ER: Endoplasmic reticulum
ERC: Endocytic recycling compartment
LDs: Lipid droplets
MCS: Membrane contact sites
OSBP: Oxysterol binding protein
Osh: OSBP homologs in yeast
PM: Plasma membrane
PS: Phosphatidylserine
STPs: Sterol transport proteins
